# Agricultural Intensification Exacerbates Spillover Effects on Soil Biogeochemistry in Adjacent Forest Remnants

**DOI:** 10.1371/journal.pone.0116474

**Published:** 2015-01-09

**Authors:** Raphael K. Didham, Gary M. Barker, Scott Bartlam, Elizabeth L. Deakin, Lisa H. Denmead, Louise M. Fisk, Jennifer M. R. Peters, Jason M. Tylianakis, Hannah R. Wright, Louis A. Schipper

**Affiliations:** 1 School of Animal Biology, The University of Western Australia, Perth, Australia; 2 CSIRO Land & Water Flagship, Perth, Australia; 3 Landcare Research, Hamilton, New Zealand; 4 Centre for Integrative Ecology, School of Biological Sciences, University of Canterbury, Christchurch, New Zealand; 5 Forests and Livelihoods Programme, CIFOR Center for International Forestry Research, Bogor, Indonesia; 6 Agroecology, Department of Crop Sciences, Georg-August-Universität Göttingen, Göttingen, Germany; 7 Institute of Agriculture, School of Earth and Environment, The University of Western Australia, Perth, Australia; 8 Science and Capability Group, Department of Conservation, Christchurch, New Zealand; 9 Department of Life Sciences, Imperial College London, Silwood Park Campus, Ascot, United Kingdom; 10 School of Science, University of Waikato, Hamilton, New Zealand; 11 Lancaster Environment Centre, Lancaster University, Lancaster, United Kingdom; USDA-ARS, UNITED STATES

## Abstract

Land-use intensification is a central element in proposed strategies to address global food security. One rationale for accepting the negative consequences of land-use intensification for farmland biodiversity is that it could ‘spare’ further expansion of agriculture into remaining natural habitats. However, in many regions of the world the only natural habitats that can be spared are fragments within landscapes dominated by agriculture. Therefore, land-sparing arguments hinge on land-use intensification having low spillover effects into adjacent protected areas, otherwise net conservation gains will diminish with increasing intensification. We test, for the first time, whether the degree of spillover from farmland into adjacent natural habitats scales in magnitude with increasing land-use intensity. We identified a continuous land-use intensity gradient across pastoral farming systems in New Zealand (based on 13 components of farmer input and soil biogeochemistry variables), and measured cumulative off-site spillover effects of fertilisers and livestock on soil biogeochemistry in 21 adjacent forest remnants. Ten of 11 measured soil properties differed significantly between remnants and intact-forest reference sites, for both fenced and unfenced remnants, at both edge and interior. For seven variables, the magnitude of effects scaled significantly with magnitude of surrounding land-use intensity, through complex interactions with fencing and edge effects. In particular, total C, total N, δ^15^N, total P and heavy-metal contaminants of phosphate fertilizers (Cd and U) increased significantly within remnants in response to increasing land-use intensity, and these effects were exacerbated in unfenced relative to fenced remnants. This suggests movement of livestock into surrounding natural habitats is a significant component of agricultural spillover, but pervasive changes in soil biogeochemistry still occur through nutrient spillover channels alone, even in fenced remnants set aside for conservation. These results have important implications for the viability of land-sparing as a strategy for balancing landscape-level conservation and production goals in agricultural landscapes.

## Introduction

One of the great challenges of the 21^st^ century is how to meet the growing food demands of a rapidly-expanding human population, while at the same time minimising environmental damage and biodiversity loss [1, 2]. Ironically, land-use intensification is increasingly seen as an important solution to this challenge [1, 3–5], even though historically it has been one of the dominant drivers of environmental degradation [6, 7]. The rationale for this is that hard trade-offs between production and the environment are unavoidable in addressing global food security, and the hope is that intensification of production on current farmland could spare further conversion of natural habitats to agriculture [4, 8]. Effectively, this ‘land sparing’ strategy is trading off local biodiversity losses against net expected biodiversity benefits at landscape, regional or global scales [3, 4, 9], as compared with ‘land sharing’ strategies that promote more environmentally-friendly farming practices with lower agricultural yield, but require a larger total land-base [4] (but see Clough et al. [10]). Of course, land sparing and land sharing are just two extremes of a continuum of competing solutions to food security issues [2, 11, 12], and the pros and cons of the two approaches have been debated extensively in the literature [3, 4, 8, 11, 13, 14].

The utility of land-sparing strategies hinges on the environmental impacts of intensification being localised and spatially independent of any biodiversity benefits accruing on set-aside land. This is true both at the landscape-context scale, which we consider here, and also at the global governance scale, within increasingly interconnected global food systems. At the landscape scale, if there are substantial off-site effects of local intensification that spill over into the surrounding land that is spared for conservation (i.e. if net conservation gains diminish with increasing intensification), then land sparing alone may not be sufficient to offset biodiversity losses. In effect, substantial spillover could severely undermine land sparing as a viable option for balancing conservation and production goals in agricultural landscapes in the future [15].

Although the potential for negative externalities from intensive farming practices has long been acknowledged by land-sparing proponents [3, 4], there has been no quantitative assessment of the degree to which spillover from farmland into adjacent natural habitats might scale in magnitude or extent with increasing land-use intensity (such as farm outputs, point- or cumulative inputs, or efficiency of output per unit input), and no critical assessment of how this might influence the land sparing versus land sharing debate. This is surprising, given that (1) agricultural practices have well-recognised negative effects on farmland biodiversity [7, 16–18], which have recently been shown to scale in magnitude with increasing land-use intensity [19], and (2) extensive cross-ecosystem spillover has been recorded from production systems into adjacent natural systems [20–23]. For example, farmer inputs of nutrients, chemicals, water and livestock are not static and can spill over from production land into adjacent natural systems [20, 24, 25], sometimes with deleterious consequences. These effects have predominantly been studied in spatially-coupled riparian and aquatic systems [26–28], but only rarely in spatially-coupled terrestrial ecosystems [20, 23, 29, 30].

In terrestrial systems, spillover from agricultural land can occur in many forms, such as the movement of generalist pests and natural enemies [21, 23, 31], the movement of nutrients from fertiliser inputs via aerial drift or down-slope leaching and run-off [20, 32, 33], and livestock encroachment into adjacent natural areas for shelter and food [34, 35]. For nutrient movement, spillover is frequently the result of inefficiency in the spatial targeting or use of on-farm inputs, and can represent a substantial resource-use efficiency gap [36]. Point estimates of the cumulative effect of these processes on natural habitat remnants embedded within intensively-managed landscapes suggest that spillover effects can be large [33, 34, 37], particularly when ecosystem remnants are small. Surprisingly, though, no studies that we are aware of have tested whether the magnitude of these terrestrial spillover effects actually scales in proportion to the magnitude of surrounding land-use intensity. If this were the case, then it could represent a crucial flaw in the underlying rationale behind land-sparing approaches [14], particularly in fine-grained mosaic landscapes where production and natural ecosystems are in close juxtaposition [38].

In this paper, we undertake the first assessment of the quantitative relationship between land-use intensification on farmland and the degree of spillover of nutrients and livestock into adjacent forest remnants. We identify a continuous land-use intensity gradient across pastoral farming systems (see also [39, 40]), reflecting the observed socio-economic and agronomic transition from low-intensity sheep farming to high-intensity dairy farming, rather than artificially comparing low versus high intensities of a single farming type (such as organic versus conventional [41], which is not a significant component of New Zealand farming systems). Instead, the rapid intensification of agricultural land-use in New Zealand has been driven by conversion to high-yielding production systems, such as dairy farming [9, 29, 42–44]. From 1960–2000 total production increased by 200–300% for major commodities with only a 6–7% increase in land area farmed [43, 45], and this trend is accelerating. Inevitably, this has fueled concern over how best to trade off sustainable commodity production with effective biodiversity conservation [9, 29, 44, 46]. On the one hand, high-yielding production systems are a major contributor to national export revenues and GDP [47], but this has come at the expense of exponential increases in the use of water, energy, fertilizer, pesticides and imported feedstocks, driving high rates of land-use intensification and environmental degradation in New Zealand [12, 42, 45, 47, 48].

Across our land-use intensity gradient, we measured the cumulative effects of nutrient and livestock spillover on soil biogeochemistry from the edge to the interior of adjacent forest remnants that had been spared from agricultural conversion. We focussed on soil biogeochemistry because soil properties and soil processes represent the foundation on which above-ground biodiversity and ecosystem functioning depend [49]. Land-use intensification is known to have had a very large influence on soil properties across agricultural land in New Zealand [50]. A regional analysis across 222 soils [50] found that most of the variance in key soil parameters, such as total nitrogen (N), carbon to nitrogen (C:N) ratio, pH, and available phosphorus (Olsen P), was explained by an over-riding influence of in situ anthropogenic land use, rather than by underlying soil type. More recently, studies have also shown that the relative isotopic ratio of ^15^N to ^14^N (δ^15^N) is a sensitive marker of cumulative anthropogenic fertilizer effects on N cycling processes [51, 52]. Across 210 New Zealand soils of varying land use, Stevenson et al. [51] found that fractionating losses were significantly greater in more intensive land-use types, resulting in substantially elevated δ^15^N on high-intensity dairy farms relative to low-intensity sheep farms or natural ecosystem remnants. Similarly, Schipper et al. [53] showed that concentrations of the heavy-metal contaminants cadmium (Cd) and uranium (U) in rock phosphate fertilisers can be used as sensitive cumulative markers of soil phosphorus dynamics. These anthropogenic markers are particularly useful because other response measures can be strongly influenced by inherent heterogeneity in the landscape prior to conversion to farming, and there can be a propensity for farming activities to be biased toward some parts of the landscape over others. By contrast, cumulative levels of Cd and U typically scale linearly with total fertiliser P inputs into the soil, and both are dramatically elevated above historical baseline levels in farmland soils [53–56], as well as in adjacent native forest remnants [37]. Despite these findings, there is very little information on the levels of agricultural inputs that might give rise to off-site environmental effects such as eutrophication of water bodies [57], or degradation of soil physical conditions in native forests [37, 58].

Here, we utilise these proven markers of anthropogenic effects on soil properties to test whether the magnitude of off-site spillover of nutrients into adjacent forest remnants scaled in proportion to agricultural intensification on farmland. We measured gradients in soil biogeochemistry changes from the edge to the interior of native forest remnants, and contrasted fenced versus unfenced fragments across the land-use intensification gradient to partition the relative influences of livestock exclusion and nutrient spillover effects from agrochemical inputs. Together, these can be used to predict, and potentially mitigate, the effects of land-use intensification on native forest remnants embedded within an agricultural mosaic.

## Methods

### Study area

**Figure 1:**
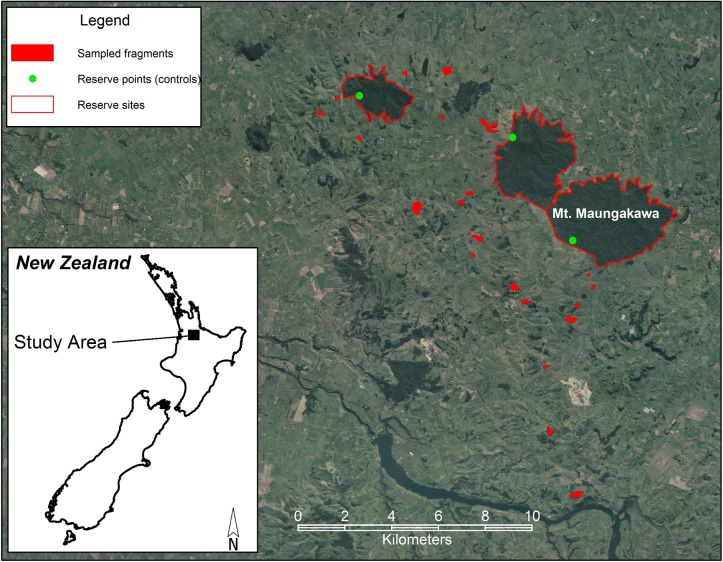
Locations of the study sites in the Waikato region of northern New Zealand. Imagery © Waikato Regional Aerial Photography Service (WRAPS) 2012. Imagery sourced from Waikato Regional Council. Licensed for re-use under the Creative Commons Attribution 3.0 New Zealand license (http://creativecommons.org/licenses/by/3.0/nz/).

The study was conducted on farmland in the Waipa District (37°49’S 175°36’E) within the Waikato Region of the North Island, New Zealand (Fig. 1). Field research carried out on private land was conducted with the permission of the landowner (land ownership data available from http://www.linz.govt.nz/). Field research carried out on public land was conducted under permit from the Department of Conservation, New Zealand. Map localities are indicated in Fig. 1. Field research did not involve endangered or protected species.

The majority of land in the Waikato Region was cleared of native forest and scrub cover, and converted to pastoral production in the early 20th century. For example, of the 145,000 ha of native forest that existed in the Waipa district prior to human settlement, only 6% now remains [59]. Because vegetation clearing was selective, most of the remaining native forest is in areas that were considered less valuable for agriculture such as gullies, steep slopes and rocky terrain [60]. Furthermore, a high proportion of remaining forest remnants are small (<5 ha) and dominated by tawa (*Beilschmiedia tawa* (A.Cunn.) Kirk) on moderately rolling hill country (100–400m a.s.l.) [61]. Grazing livestock (predominantly cattle and sheep) have had unrestricted access to most forest remnants, but in the last 10–20 years it has become increasingly common for farmers to fence remnant vegetation to exclude livestock. The Waikato region follows the general trends seen in the rest of New Zealand over the past 40 years, with rapidly increasing intensification of land-use and a shift to higher input-higher output farming systems, dominated by dairy farming [42, 45, 62].

### Study design

The Land Environments of New Zealand (LENZ) classification [63] was used to identify forest remnants across the Waipa District with similar geomorphology and vegetation cover (i.e. tawa forest on rolling hill country between 100–400m a.s.l.). Spatial data layers were then created in geographic information systems software (ArcGIS version 9) to select candidate forest remnants to visit (n = 50) which had a slope of <25° and remnant area >1 ha. Site visits and interviews with the landowners were carried out to further determine the suitability of individual forest remnants for the project, based on a set of objective criteria including landowner permission, livestock exclusion (unfenced or fenced for a minimum of 10 years), slope in the sampling zone (<25°), and accessibility of pasture upslope of the remnant (to maintain consistency among sites in potential overland flows of nutrients). From this pool of suitable remnants, fenced and unfenced remnants were randomly selected to achieve a stratified distributed across farms with a wide range of farming types likely to vary in land-use intensity (i.e. as random selection proceeded, remnants were rejected if this would have led to over-representation of particular farming types). This process resulted in 11 fenced and 10 unfenced forest remnants being selected for study (Fig. 1). Landowners were then asked to provide more detailed information relating to their farming practices, including information on animal stocking rates, fertiliser inputs and the use of forest remnants for stock grazing and shelter.

Three large forest reserves in the same region were selected as reference sites against which to compare the study remnants. These were Te Miro Scenic Reserve (403 ha), Maungakawa Reserve (965 ha) and Te Tapui Reserve (1377 ha) (Fig. 1). Compared with the study remnants, the sampling points within reference sites were similar in elevation, soil type and vegetation type, but were relatively unaffected by adjacent agricultural disturbances. While they were not ‘pristine’ they represent the best examples of relatively unmodified tawa forest left in the region.

For the 21 forest remnants and three reference sites, we first (1) quantified the (relative) agricultural land-use intensity on farmland surrounding each forest site, and then (2) tested whether this measure of land-use intensity was a significant predictor of the magnitude of nutrient spillover into adjacent forest remnants.

### Quantifying multiple components of land-use intensity across farms

On each farm we measured 13 components of land-use intensity, consisting of four short- to medium-term farmer input variables (i.e. N input, P input, lime input, and stocking rate) and nine medium- to long-term soil biogeochemistry variables (pH, Olsen P, total C, total N, C:N ratio, δ^15^N, total P, total Cd, and total U), and subsequently used these to create a composite index of land-use intensity in a principal components analysis (PCA), as detailed below. For our study system there was no quantitative information available on variation in farm inputs versus outputs through time, from which to estimate land-use intensity in terms of absolute yield, yield gaps or resource-use efficiency gaps in a direct manner [36]. Instead, we adopt the pragmatic reasoning of Kleijn et al. (2009) that inputs are more likely to be related to ecological impacts than yield per se [19]. To validate the utility of our composite land-use intensity index, based on both short-term inputs and longer-term markers of the inefficiency of spatial targeting of inputs, we conducted multiple sensitivity tests of index performance in subsequent analyses.

**Four farmer input measures**. Although no detailed long-term records of land-use inputs were available for the farms we studied, we conducted semi-structured interviews with farmers to gather information on recent farming practices on the land immediately surrounding our 21 study remnants. We asked farmers for details of the quantity and frequency of application of all types of fertiliser and lime, and a detailed history of stocking rates in the pasture immediately upslope of each forest remnant. Given the lack of written farm records in most cases, and changes in land ownership and farming practices over time, we consider that these farmer input measures reliably reflect recent land-use practices within a period of approximately five years.

Recent N input was calculated from a combination of N-fertiliser application and model-estimated N fixation rates by the legume component of the pastures (primarily *Trifolium repens* L.). Two N-containing fertilisers, urea (N:P:K:S = 46:0:0:0) and DAP (Di-Ammonium Phosphate; 18:20:0:1), were used recently on paddocks adjacent to 8 of our 21 forest remnants, via either ground-level dispersal (by tractors or trucks) or aerial dispersal (by crop-dusting aircraft). Across our entire sample of sites, application rates varied from 0–96 kg N per ha per year. On-farm N fixation rates were estimated from OVERSEER version 5.4 farm nutrient-budget model [64, 65] based on estimated parameters for topography, climate, geomorphology, pasture type and land management practices at each site. Estimated N fixation rates varied from 37–151 kg N per ha per year. New Zealand is known to be somewhat unusual in comparison with other temperate countries in that biological N fixation makes up such a large part of the N budget of grazed pastures [65].

Recent P input was calculated from application of the P-containing fertilisers superphosphate (N:P:K:S = 0:9:0:11), RPR (Reactive phosphate rock; 0:14:0:0) or DAP (18:20:0:1) to paddocks surrounding our forest remnant sites. Application was via either ground-level or aerial dispersal, and varied from 0–50 kg P per ha per year.

Lime was applied to paddocks surrounding 8 of our 21 study sites over the preceding 5 years, at average annual rates of 200–700 kg per ha per yr, via either ground-level or aerial dispersal.

‘Stocking rate’ is a standardised farmer measure of livestock density per unit area of land in New Zealand, calculated by converting the type of stock (for example, sheep, deer, beef cattle or dairy cattle), and their sex and breeding status, to common stock units (“ewe equivalents” in SU/ha; S1 Table). In some cases farmers were able to specify stocking rate directly in standardised stock units. In other cases, where the numbers, sexes and breeding status of individual animals of each livestock species were specified, we calculated stock unit equivalents from S1 Table and divided by total farm area. Where only a rough average density of livestock species was provided (e.g., “3 dairy cows per ha”), we applied ‘typical’ reference values for the percent individuals of different sexes and breeding status in the Waikato region (S1 Table). Stocking rates varied from 6.9–18.9 SU/ha across farms (S2 Table).

**Nine soil biogeochemistry measures**. Farmers alter short-term nutrient and livestock inputs to a paddock in response to the status of soil biogeochemistry and pasture productivity. In turn, soil biogeochemistry is responsive in the medium- to long-term to cumulative farmer inputs. Of the soil biogeochemistry variables measured, we considered pH and available P (Olsen P) to be responsive to changing land-use practices over comparatively short timeframes [66, 67], whereas we considered that total C, total N, C:N ratio, δ^15^N, total P, total Cd, and total U would be responsive over much longer timeframes [51–53, 56, 68], and therefore better reflect the cumulative historical influence of land-use intensity rather than very recent changes in land-use practice.

Soil biogeochemistry was measured in open pasture 46.5 m upslope from each forest remnant by collecting 18 soil cores of 2 cm in diameter down to a depth of 100 mm at randomly-selected points along a 20 m sampling line running parallel to the remnant edge. From these 18 cores, six were randomly allocated to each of three pooled samples so as to minimise the effect of spatial heterogeneity across individual cores, while taking into account the logistical constraints of analytical costs. The pooled soil samples were passed through a 6 mm mesh sieve, air-dried to constant weight and stored dry in plastic pottles in a cool, dark room until further analysis.

To measure pH, a subsample of 10 g of air-dried soil was mixed with 25 ml of deionised water (1:2.5 soil to water ratio) then left to settle overnight. The next day the pH of the supernatant was measured with a combination electrode, and varied from 4.65–5.79 across farms.

Total C and total N were analysed on the air-dried ground soil samples, using a LECO TruSpec CN Carbon/Nitrogen Determinator (TruSpec, St Joseph, MI, USA), and corrected for soil moisture factor and bulk density (see below). Total C varied from 31.151–83.408 mg.cm^-3^, total N varied from 3.119–8.371 mg.cm^-3^ and the ratio of C:N varied from 8.469–11.021 across farms.

The stable isotope composition of nitrogen in the soil was analysed on the air-dried ground samples using a Dumas elemental analyser (Europa Scientific ANCA-SL) interfaced with a Europa Scientific 20–20 Stable Isotope Analyser. Results are reported in the δ^15^N notation:

$$\delta^{15}N\;=\;\left[R_{sample}/R_{std—}1\right]\;\times \;1000$$

where R_sample_ is the ^15^N/^14^N ratio of the sample and R_std_ is the ^15^N/^14^N ratio of the laboratory standard (urea), which was calibrated against a certified standard (atmospheric nitrogen). Heavy nitrogen enrichment, δ^15^N, has units of parts per thousand (‰), and varied from 4.093–7.630 ‰ across farms.

Phosphorus is continually cycled through soils, plants and organisms, and the Olsen P method measures the labile pool of available P [69]. The Olsen P method gives good correlations with P uptake by plants in New Zealand [70], and is therefore a good indicator of plant response to available P. The method used here was that of the Australasian Soil and Plant Analysis Council (ASPAC), modified for application to soils of the Pacific region [71], based on a large body of earlier research [69, 70, 72, 73].

We mixed 1.00 g ± 0.01 g of air-dried soil with 20 ml of 0.5 M NaHCO_3_ (pH 8.5) for 30 minutes in an end-over-end shaker, then filtered through Whatman no. 42 filter paper and stored the filtrate in the refrigerator overnight. We added 10 ml of 0.1 M H_2_SO_4_ to 4 ml of the filtrate to neutralize it, and then left it to stand for one to two hours with intermittent shaking to ensure complete reaction. Subsequently, 4 ml of Murphy and Riley Reagent B was added, the samples mixed well, and left for approximately one hour for colour production (Murphy and Riley Reagent B is 1.056 g ascorbic acid added to 100 ml of Murphy and Riley Reagent A, where Reagent A is a 1.2% solution of ammonium molybdate with 0.1 mg.ml^-1^ antimony in 2.5 M H_2_SO_4_). Absorbance was read at 880 nm. Olsen P was calculated from a standard curve of the absorbances of 0, 0.4, 1, 2, 4, 6 and 8 mg.L^-1^ P standards, taking into account the moisture factor of the air-dried soil and bulk density of the sample. Olsen P varied from 1.3–55.1 μg.cm^-3^ across farms.

To measure total P, total Cd, and total U we carried out an acid-reflux extraction of 1.00 g ± 0.01 g of air-dried ground soil in a solution of 4 ml (1+1) HNO_3_ and 10 ml (1+4) HCl at 95°C for 30 minutes. The resulting solution was centrifuged, diluted 1:4 with Type I deionised water, and the HNO_3_ concentration adjusted to 2%. The solution was then passed through 0.45 µm filter units and analysed for total recoverable elements, including P, Cd and U, by inductively coupled plasma—mass spectrometry (ICP-MS) [74]. Values were corrected for soil moisture factor and bulk density. Total P varied from 351–2014 μg.cm^-3^, total Cd varied from 0.25–0.84 μg.cm^-3^ and total U varied from 0.88–2.10 μg.cm^-3^ across farms.

**Correction for bulk density and moisture factor**. Measures of soil biogeochemistry were corrected for variation in bulk density and moisture factor among soils on different farms. We measured bulk density in open pasture 46.5 m upslope from each forest remnant by collecting three soil cores of 9.8 cm in diameter to a depth of 100 mm at randomly-selected points along the same sampling line as the nutrient cores were taken. Litter, loose organic matter, grass, and grass roots were not included in the cores, making their effective depth range approximately 25 to 100 mm. For each of these bulk density cores, soil was dried at 105°C until a constant weight was achieved. The average of the three values was taken as a paddock-scale measure of bulk density for each site, and varied from 0.5–1.0 g.cm^-3^ across farms.

For analyses that required the use of air-dried rather than oven-dried soils we calculated a soil moisture factor for each sample, so that we could account for differences in soil moisture-holding capacity and variation in moisture content of air-dried soil with time and storage conditions [72]. For each sample, a 20 g subsample of air-dried soil was extracted using a multiple-slot sample divider (riffle box). Each subsample was then hand-ground to a fine powder using an agate mortar and pestle. From this subsample, 4 g was weighed out and dried to a constant weight in a 105°C oven, and then reweighed. Moisture factor equals the moist (air-dried) weight divided by the oven-dried weight.

### Sampling soil biogeochemistry gradients within forest remnants

Within each of the 21 forest remnants, a 20 m wide × 46.5 m long zone was marked out perpendicular to the forest edge, in which all soil sampling was conducted. This sampling zone was directly down slope from the pasture measurements described above. In this zone, five sampling points were established on a log_3_ distance scale from the forest edge to the forest interior at 3^0^, 3^1^, 3^2^, 3^3^ and 3^3.5^ m (i.e. 0, 3, 9, 27 and 46.5 m). The edge (0 m) was defined as the edge of the forest leaf-litter accumulation zone, which in most cases was the canopy drip line [75], but in some fenced remnants where the drip line was outside of the fence then ploughing and grazing meant that the leaf-litter accumulation zone corresponded to the fence line instead. The maximum distance sampled along the edge gradient was chosen to reflect the maximum log_3_ distance available in the smallest remnants (i.e. 46.5 m).

The same sampling design was employed in the three reference forest sites, except that the log_3_ edge gradient was extended to eight sampling points, including 3^4^, 3^5^ and 3^5.5^ m inside the forest (i.e. 81 m, 243 m and 420 m), in order to better describe interior forest conditions. Here, we use the 243 m and 420 m values, combined, as our comparative ‘reference’ conditions.

Along each of the edge gradients, soil samples were collected in an identical manner to those collected in the adjacent open pasture sites (as described above). At each distance from edge we collected three bulk density cores (9.8 cm diameter) and 18 soil nutrient cores (2 cm diameter) to a depth of 100 mm at randomly-selected points along a 20 m sampling line running parallel to the remnant edge. The 18 soil nutrient cores were randomly allocated to one of three pooled samples, each of which was analysed separately for soil moisture and for the nine soil biogeochemistry measures, pH, total C, total N, C:N ratio, δ^15^N, Olsen P, total P, total Cd, and total U, using the standard laboratory protocols detailed above.

### Statistical Analyses

**Quantifying a land-use intensity gradient across farms**. We had no *a priori* evidence for a single variable that would represent an ‘ideal’ indicator of land-use intensity across farms, and no objective means of determining the relative weighting of multiple collinear predictors (S1 Fig.). Therefore, we constructed an unweighted composite index of land-use intensity across farms, based on our 13 farmer input and soil biogeochemistry measures (c.f. [40]), using a correlation-based Principle Components Analysis (PCA) conducted in Primer v.6 [76, 77]. Farmer N input, lime input, Olsen P, total P and total Cd were log_e_-transformed, and farmer P input was reverse transformed (-log_e_ (x_max_-x)) to approximate normality, and minimise the effects of strong outliers, followed by normalisation of variables to account for the different unit scales of measure [77].

**Sensitivity analyses to validate the composite land-use intensity gradient**. We conducted three sensitivity analyses to test for potential confounding influences on our interpretation of the composite PCA gradient of land-use intensity. First, we tested the validity of using both short-term and longer-term measures of land-use intensity in our single composite PCA index. Our *a priori* expectation was that the four farmer-input measures calculated for the preceding 5 years (N input, P input, lime input, and stocking rate) as well as two labile soil biogeochemistry measures (pH and Olsen P) would reflect comparatively short-term land-use change, while the remaining seven soil biogeochemistry measures (total C, total N, C:N ratio, δ^15^N, total P, total Cd, and total U) would reflect longer-term historical land-use effects. We conducted a sensitivity test of the degree of consensus between land-use intensity based on a PCA ordination of the six ‘recent’ land-use measures, versus a separate PCA ordination based on the seven ‘historical’ land-use measures. We correlated relative site-to-site dissimilarity in ordination space between the two approaches, first using a parametric Pearson correlation between recent versus historical land-use intensity based on PCA axis 1 scores alone, and second using a non-parametric rank correlation based on the full Euclidean dissimilarity matrix using the ‘RELATE’ function in Primer v6 (all five PCA axes at once). Deviation from strict concordance between short-term versus long-term measures of land-use intensity was dealt with by calculating the residuals around the recent-versus-historical land-use relationship as an index of ‘recent change in intensity’ (i.e. any mis-match in relative site rankings that might indicate higher or lower recent farm inputs than might otherwise be expected from the longer-term legacy of soil nutrient inputs). We confirmed that there was no significant correlation between ‘recent change in intensity’ and the original composite index of land-use intensity based on all 13 farmer input and soil biogeochemistry measures (see S1 Supporting Information for further details).

Second, due to the wide geographic spread of study sites (Fig. 1) it was not possible to constrain all farms to a single soil type. The study sites spanned three soil orders/groups in the NZ Soil Classification (NZSC) scheme, split predominantly between Typic Orthic Brown Soils (12 sites) and Typic Orthic Allophanic Soils (6 sites), with fewer sites having Mottled Orthic Recent Soils (3 sites) (S3 Table). Different soil types are known to have different nutrient retention properties, drainage, pH buffering capacity and susceptibility to land-use effects [78]. Therefore, we used a non-parametric Kruskal-Wallis rank sum test to assess whether the composite PCA measure of land-use intensity varied significantly between soil orders/groups (see S1 Supporting Information for further details).

Third, there were several instances where paddocks surrounding study remnants were located on the same farm, so we tested for potential confounding spatial autocorrelation of PCA axis 1 land-use intensity values using a spline correlogram to estimate the spatial dependence of the axis scores as a continuous function of distance (rather than in binned distance classes, such as in a Mantel Correlogram). This was estimated using the spline.correlog function in the ‘ncf’ package in R 2.14.2 [79].

**Testing the impact of surrounding land-use intensity on soil biogeochemistry inside forest remnants**. We tested the effects of surrounding agricultural land-use intensity on edge-to-interior gradients in soil biogeochemistry within the 21 forest remnants using linear mixed models (LMMs) in the ‘nlme’ package [80] in R version 3.0.2 [81]. Pasture slope (i.e. the average slope of agricultural land adjacent to the forest remnant), forest slope (i.e. the average slope of the forest remnant within the sampling zone), the logarithm of patch area, and the index of ‘recent change in intensity’ were specified as fixed covariate effects (S4 Table), to remove these sources of variation before testing our variables of interest. Land-use intensity (PCA axis 1 scores; S4 Table), fencing category (fenced versus unfenced), distance from edge (second-order polynomial function of log_3_ distance from edge) and their interactions were specified as fixed treatment effects. Random intercepts were specified for distance-from-edge (‘distance block within site’) nested within forest remnants (‘site’), to account for non-independence of samples. A random second-order polynomial slope was specified to account for stochastic variation in edge effects across sites (‘random edge effect’) [82].

For each of 11 response variables (bulk density, soil moisture, pH, total C, total N, C:N ratio, δ^15^N, Olsen P, total P, total Cd, and total U), we first converted variates to standardised differentials relative to reference forest conditions, by subtracting the mean of the three samples taken at each of the 243 m and 420 m distances within each of the three reference forest sites (n = 18). The purpose of this was to provide a direct indication of whether model coefficients were significantly different from average values expected at the interior of the separate reference sites (i.e. the most ‘pristine’ condition available). For each response variable, we then tested the residuals of the full models for normality and homogeneity of variances. Olsen P and total P required log_e_-transformation to meet the assumption of normality of residuals, and the soil moisture model required unequal variance structure to be fitted using the varIdent function in nlme in order to deal with heteroscedasticity.

Model simplification was performed using a multi-model inference approach in the MuMIn package in R [83]. We constructed competing models representing all possible subsets of fixed effects (with predictor variables centred and standardised by 2 S.D. [84]), and compared their relative fit based on maximum likelihood (ML) estimation, and Akaike Information Criterion (AICc) corrected for small-sample bias [85]. From the candidate set of ‘top models’ within 2 AICc units of the best-fit model, we estimated the final model coefficients using model averaging and restricted maximum likelihood (REML) estimation [86]. A model averaging approach accounts for model selection uncertainty by using the weighted averages of parameter estimates based on relative AICc model support. Models that contribute little information on variance in response are given correspondingly lower weight [86].

For the final model-averaged predictor set, we used the approach of Nakagawa and Schielzeth [87] to estimate absolute model fit using marginal $R_{GLMM}^{2}$ (variance explained by just the fixed effects) and conditional $R_{GLMM}^{2}$ (variance explained by both fixed and random effects). In addition, to inspect how the inclusion of the fixed effects in the model reduced (or increased) variance components at different levels of the mixed-model, we calculated the percent change in variance (PCV) for each level based on a comparison of the final model versus a null (intercept-only) model that contained the same random effects [87].

## Results

### Quantifying a land-use intensity gradient across farms

**Figure 2:**
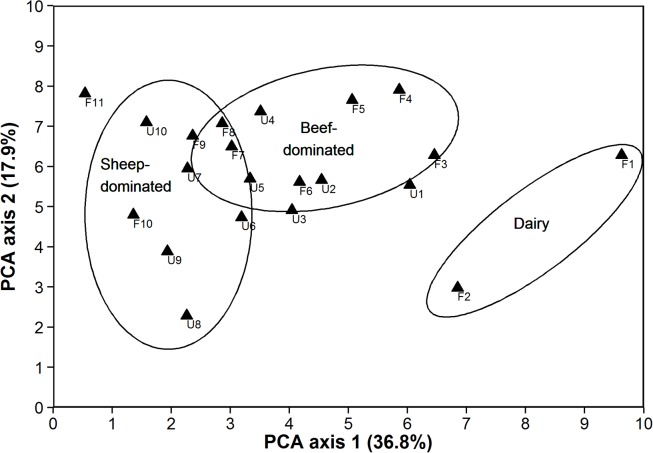
Biplot of PCA axes 1 and 2 showing the dominant gradients of variation in land-use intensity across farms, based on four short-term farmer input measures and nine longer-term soil biogeochemistry measures (see S2 Fig. for factor loadings). Land-use intensity increased (left to right) with the change in type of farming from sheep-dominated to beef-dominated to dairy-dominated. Note that all sheep-dominated farms had a mix of livestock types, including sheep, beef and dairy grazing, whereas most beef-dominated farms did not have sheep (see S2 Table for a breakdown of stocking rates per livestock class). Ellipses are for illustrative purposes only.

**Figure 3:**
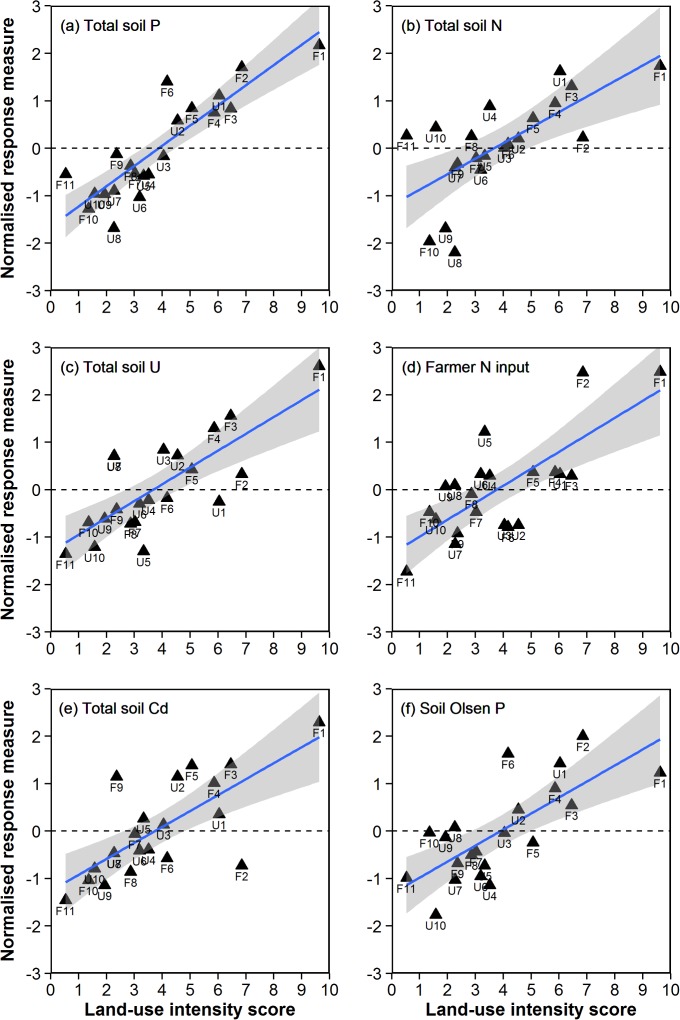
Relationships between the composite PCA axis 1 measure of land-use intensity (see Fig. 2) and the six component variables with the highest factor loadings (S5 Table, S2 Fig.), including both soil biogeochemistry and farmer input measures.

The PCA ordination based on all 13 measures of farmer inputs and soil biogeochemistry (Table 1) identified a strong gradient of variation in agricultural land-use intensity across farms (Fig. 2). A 2-d representation of the 13-d dataset (Fig. 2) explained 54.7% of the variation in farmer inputs and soil biogeochemistry across sites, and described a dominant gradient along PCA axis 1 from lower-intensity sheep- and beef-dominated farms to higher-intensity dairy farms (Fig. 2, S2 Table). The eigenvectors (S5 Table) and factor loadings (S2 Fig.) for PCA axis 1 indicated that this gradient was characterised by high cumulative measures of total N, total P, total U and total Cd in the soil, as well as high recent farmer N inputs and high Olsen P values in dairy farms compared to mixed sheep farms (Fig. 3).

**Table 1:** Components of variation in agricultural land-use intensity on farms surrounding the 21 forest remnants.

**Site code**	**Farm area ha**	**Bulk density g.cm^-3^**	**Moisture factor**	**N input kg.ha^-1^. yr^-1^**	**P input kg.ha^-1^. yr^-1^**	**Lime input t.ha^-1^.yr^-1^**	**Stocking rate SU.ha^-1^**	**pH**	**Olsen P μg.cm^-3^**	**Total C mg.cm^-3^**	**Total N mg.cm^-3^**	**C:N ratio**	**δ^15^N ‰**	**Total P μg.cm^-3^**	**Total Cd μg.cm^-3^**	**Total U μg.cm^-3^**
F1	120	0.702	1.107	216.0	50.0	0.0	18.8	5.30	24.31	74.64	8.11	9.21	5.52	1879.24	0.82	2.07
F2	150	0.660	1.092	214.8	36.4	0.0	18.9	4.85	45.87	59.26	6.31	9.40	6.85	1554.14	0.31	1.42
F3	560^a^	0.677	1.121	95.8	31.9	0.0	12.0	4.93	13.84	79.97	7.60	10.52	5.64	1090.82	0.62	1.77
F4	270^e^	0.689	1.081	98.6	31.9	0.0	14.3	5.74	18.50	75.14	7.17	10.48	4.46	1054.46	0.54	1.70
F5	270^e^	0.647	1.088	98.6	31.9	0.0	14.3	5.57	7.21	67.04	6.80	9.86	4.40	1094.07	0.61	1.45
F6	120	0.618	1.114	64.0	27.3	0.0	10.6	5.18	33.80	57.20	6.15	9.30	5.80	1375.08	0.33	1.28
F7	404^d^	0.627	1.086	72.0	43.4	0.0	12.5	5.37	6.20	55.38	5.79	9.56	4.65	624.19	0.39	1.14
F8	330	0.640	1.108	83.0	31.9	0.7	14.6	5.62	5.83	64.62	6.34	10.19	4.34	666.16	0.30	1.13
F9	121	0.690	1.102	61.0	36.4	2.5	10.0	5.65	5.06	48.30	5.65	8.54	5.93	736.79	0.57	1.22
F10	404^d^	0.946	1.055	72.0	37.2	0.0	12.5	5.28	8.58	35.37	3.70	9.55	4.44	460.98	0.28	1.14
F11	31	0.590	1.117	45.1	1.1	0.0	6.9	5.49	3.93	54.81	6.36	8.62	4.47	621.22	0.25	0.95
U1	200^c^	0.657	1.095	97.0	22.8	0.0	17.5	5.07	28.60	70.96	7.97	8.90	6.15	1218.93	0.44	1.26
U2	404^b^	0.858	1.094	65.0	22.8	0.2	12.4	5.24	12.77	58.59	6.29	9.43	6.61	985.06	0.57	1.54
U3	404^b^	0.707	1.109	65.0	22.8	0.2	12.4	5.13	8.55	63.05	6.05	10.42	7.30	723.75	0.41	1.57
U4	560^a^	0.643	1.114	95.8	31.9	0.0	12.0	5.53	3.45	70.21	7.10	9.89	5.30	618.72	0.35	1.27
U5	275	0.546	1.109	135.0	45.0	1.0	11.3	5.24	4.86	59.01	5.85	10.09	5.62	611.87	0.43	0.97
U6	200^c^	0.706	1.125	97.0	22.8	0.0	17.5	5.15	4.02	57.27	5.50	10.40	5.73	509.67	0.35	1.25
U7	180	0.799	1.081	56.0	34.1	0.0	9.6	5.20	3.77	49.63	5.56	8.92	5.45	538.46	0.34	1.53
U8	120	0.996	1.048	98.0	23.7	0.6	16.0	4.96	9.44	32.76	3.42	9.58	6.77	389.98	0.34	1.53
U9	192	0.782	1.058	88.0	22.8	0.5	15.6	5.06	7.91	43.69	4.03	10.83	4.89	522.30	0.28	1.16
U10	357	0.590	1.121	68.0	0.0	0.0	11.1	5.32	2.06	64.56	6.56	9.84	4.93	524.15	0.31	0.99

‘F’ and ‘U’ site codes indicate whether the adjacent forest remnants were fenced or unfenced, respectively, but this information had no bearing on the calculation of land-use intensity. Values for ‘farm area’ that share a common superscript denote paddocks that were located on the same farm, but were at least 420 m apart. All soil measures are the average of three recorded values taken at each site.

Sites with fenced versus unfenced forest remnants were not uniformly distributed across PCA axis 1 (Fig. 2), with farms surrounding fenced remnants having a much wider variation in farmer inputs and soil biogeochemistry values compared with farms surrounding unfenced remnants (Fig. 3).

Site dissimilarity along PCA axis 2 was relatively low among the majority of sites, and would have been negligible except for a few notable outliers (e.g., F2, F10, U8, U9; Fig. 2) with unusually low soil pH, low soil N and C, but high stocking rate and high soil δ^15^N relative to other farms (S5 Table, S2 Fig.).

### Sensitivity analyses to validate the composite land-use intensity gradient

**High correlation among short-term versus long-term measures of land-use intensity**. In the first of three sensitivity tests, a PCA based on six ‘recent’ land-use measures provided the same ordering of sites as a PCA based on seven ‘historical’ land-use measures, and the two were found to be highly correlated in their relative site dissimilarity in ordination space (S3 Fig.). There was a significant positive correlation between recent versus historical land-use intensity based on PCA axis 1 scores (Pearson’s correlation, r = 0.49, p = 0.025; S3a Fig.), and similarly a significant positive correlation based on the full Euclidean dissimilarity matrix using the ‘RELATE’ function in Primer v6 (Spearman’s Rho = 0.231, P = 0.041; 9999 random permutations). Outlier sites identified in this analysis (sites below the diagonal line in S3a Fig.) were the same outlier sites identified in the composite land-use intensity gradient in Fig. 2, confirming a partial mis-match in evidence based on short-term versus long-term measures in these four cases. Using the residuals around the recent-versus-historical land-use relationship in S3a Fig. as an index of ‘recent change in intensity’ (S4 Table), we confirmed that there was no significant correlation between ‘recent change in intensity’ and the original composite index of land-use intensity (Pearson’s correlation r = −0.25, p = 0.274; S3b Fig.); i.e., ‘recent intensification’ does not co-vary with absolute intensity across farms. Finally, we confirmed that a correction for ‘recent change in intensity’ had minimal effect on the rank-ordering of sites along the composite PCA axis 1 land-use intensity gradient (see S1 Supporting Information, S3c Fig.).

**No confounding effect of variation in soil type classification across sites**. We found no evidence that the land-use intensity gradient was influenced by variation in soil type (Kruskal-Wallis rank sum test for differences in PCA axis 1 scores among the three soil groups: χ^2^ = 1.537, d.f. = 2, P = 0.464; or among the six soil families: χ^2^ = 3.154, d.f. = 5, P = 0.676; S4 Fig.). Although P retention is known to differ strongly among soil types, we found no evidence that farm P availability (Olsen P) co-varied with soil type (among soil groups: χ^2^ = 0.234, d.f. = 2, P = 0.890; or among soil families: χ^2^ = 5.848, d.f. = 5, P = 0.321). Similarly, pH buffering capacity is known to differ strongly among soil types, and in this case we did find a small but significant effect of soil type on farmer lime input (among soil groups: χ^2^ = 6.382, d.f. = 2, P = 0.041; although not among soil families: χ^2^ = 10.163, d.f. = 5, P = 0.071). Although this effect should be interpreted with caution, given the increased probability of Type I errors from multiple tests, it appears that farmers at the three sites with Mottled Orthic Recent Soils tended to apply more lime, whereas farmers on Orthic Allophanic soils applied particularly little lime in the preceding five years (see S1 Supporting Information). Despite this, the recent history of farmer lime input did not influence covariance between soil type and soil pH across paddocks (among soil groups: χ^2^ = 2.037, d.f. = 2, P = 0.361; or among soil families: χ^2^ = 5.291, d.f. = 5, P = 0.381). Finally, we tested for covariance between soil type and the index of ‘recent change in intensity’, but found no significant relationship (among soil groups: χ^2^ = 1.649, d.f. = 2, P = 0.438; or among soil families: χ^2^ = 4.527, d.f. = 5, P = 0.476).

**No spatial autocorrelation of land-use intensity across farms**. In the spline correlogram (S5 Fig.), there was no evidence that paddocks that were closer together in space (e.g., on the same farm, or adjacent farms) had land-use intensity scores that were significantly more similar than expected by chance alone.

From these sensitivity analyses, we concluded that the gradient of PCA axis 1 scores based on all 13 land-use measures was a robust, general measure of agricultural intensity across farms. We confirmed that land-use intensity surrounding the three reference sites was typical of average land-use intensity values on farms surrounding the 21 forest remnants (see S1 Supporting Information; S6 Table), with none of the farms adjacent to reference sites having high leverage in the analysis (S6 Fig.).

### Testing the impact of surrounding land-use intensity on soil biogeochemistry inside forest remnants

Prior to testing the effects of agricultural land-use intensity on soil biogeochemistry in forest remnants, we tested for potential confounding variation in soil moisture factor and bulk density across remnants, and found no significant effect of the land-use intensity gradient on either variable (Table 2). In the case of the soil moisture factor, there was high site-to-site variability (range 1.032–1.244), but the model-average set included the null (intercept-only) model and there was no evidence that soil moisture differed significantly from forest reference conditions (i.e. the model intercept did not differ significantly from zero) or that land-use, fencing treatment or edge effects significantly influenced the model-averaged coefficients (Table 2, S7 Fig.).

**Table 2:** Results of mixed-effects modelling of the effects of land-use intensity on soil nutrient geochemistry in native forest remnants embedded within production landscapes.

**Response variable:**	**Soil moisture**		**Bulk density**		**pH**		**Total C**	
	**Null model**	**Full model**	**Null model**	**Full model**	**Null model**	**Full model**	**Null model**	**Full model**
Fixed effects	*b* [±1 SE]	*b* [±1 SE]	*b* [±1 SE]	*b* [±1 SE]	*b* [±1 SE]	*b* [±1 SE]	*b* [±1 SE]	*b* [±1 SE]
Intercept	-0.009 [0.005]	-0.009 [0.005]	**0.161 [0.036]**	**0.232 [0.038]**	**0.267 [0.057]**	**0.245 [0.050]**	-2.852 [2.187]	6.751 [3.777]
Pasture slope	-	-0.013 [0.010]	-	**0.116 [0.054]**	-	**0.202 [0.095]**	-	-
Forest slope	-	-0.008 [0.010]	-	0.063 [0.051]	-	**-0.191 [0.088]**	-	-
Patch area	-	0.013 [0.011]	-	-	-	0.175 [0.092]	-	**7.470 [3.443]**
Recent change in intensity	-	-0.020 [0.010]	-	**0.156 [0.054]**	-	**-0.257 [0.092]**	-	-
Land-use intensity gradient	-	-	-	-	-	**-0.202 [0.087]**	-	24.708 [12.460]
Fencing	-	-	-	**-0.137 [0.052]**	-	0.075 [0.086]	-	**-12.692 [3.843]**
Distance from edge (linear)	-	-	-	0.034 [0.022]	-	-	-	-3.836 [2.142]
Distance from edge (quadratic)	-	-	-	0.060 [0.036]	-	-	-	-6.289 [5.176]
Land-use: Fencing	-	-	-	-	-	-	-	-24.37 [12.275]
Land-use: Distance (linear)	-	-	-	-	-	-	-	-13.933 [7.595]
Land-use: Distance (quad.)	-	-	-	-	-	-	-	-30.79 [19.701]
Fencing: Distance (linear)	-	-	-	-	-	-	-	-4.330 [3.358]
Fencing: Distance (quad)	-	-	-	-	-	-	-	-3.670 [8.710]
Land-use: Fencing: Dist(lin)	-	-	-	-	-	-	-	**24.548 [8.420]**
Land-use: Fencing:Dist(quad)	-	-	-	-	-	-	-	41.541 [21.842]
VC for random effects	VC	VC	VC	VC	VC	VC	VC	VC
Site	0.00103	0.00094	0.02820	0.01882	0.14009	0.08802	164.67164	119.73558
Random edge effect	0.00089	0.00089	0.01957	0.01863	0.13136	0.12915	241.42407	199.58430
Distance block within site	0.00004	0.00004	0.00065	0.00065	0.01987	0.01992	41.11794	41.06110
Residuals	0.00008	0.00008	0.00793	0.00794	0.02303	0.02303	36.59037	36.61568
VC for fixed effects	-	0.00009	-	0.01443	-	0.03433	-	51.08864
PCV_[Site]_	-	8.41%	-	33.26%	-	37.17%	-	27.29%
PCV_[Edge.Slope]_	-	0.48%	-	4.80%	-	1.68%	-	17.33%
PCV_[Edge.Block]_	-	7.07%	-	0.57%	-	-0.25%	-	0.14%
PCV_[Residuals]_	-	1.37%	-	-0.08%	-	.00%	-	-0.07%
$R_{GLMM(m)}^{2}$	-	4.22%	-	23.87%	-	11.75%	-	11.74%
$R_{GLMM(c)}^{2}$	-	96.15%	-	86.88%	-	92.14%	-	91.74%
AIC	-1919.447	-1920.107	-476.446	-489.806	-56.738	-65.614	2261.147	2257.928
**Response variable:**	**Total N**		**C:N ratio**		**δ^15^N**		**Olsen P**	
	**Null model**	**Full model**	**Null model**	**Full model**	**Null model**	**Full model**	**Null model**	**Full model**
Fixed effects	*b* [±1 SE]	*b* [±1 SE]	*b* [±1 SE]	*b* [±1 SE]	*b* [±1 SE]	*b* [±1 SE]	*b* [±1 SE]	*b* [±1 SE]
Intercept	0.197 [0.171]	**1.280 [0.277]**	**-2.158 [0.175]**	**-2.144 [0.237]**	**0.428 [0.175]**	0.357 [0.236]	**2.366 [0.232]**	**3.071 [0.267]**
Pasture slope		-	-	0.592 [0.327]	-	-0.481 [0.243]	-	0.344 [0.292**]**
Forest slope		-0.202 [0.260]	-	0.182 [0.324]	-	-	-	**0.828 [0.285]**
Patch area		0.428 [0.283]	-	0.185 [0.320]	-	**-0.773 [0.301]**	-	-
Recent change in intensity	-	-0.239 [0.267]	-	-0.373 [0.343]	-	0.419 [0.307]	-	**1.159 [0.298]**
Land-use intensity gradient	-	**1.047 [0.485]**	-	-	-	-0.355 [0.458]	-	-
Fencing	-	**-1.043 [0.286]**	-	0.364 [0.393]	-	-0.443 [0.283]	-	**-0.918 [0.362]**
Distance from edge (linear)	-	**-0.524 [0.161]**	-	0.238 [0.205]	-	**-0.667 [0.133]**	-	-0.468 [0.279]
Distance from edge (quadratic)	-	-0.399 [0.312]	-	-0.329 [0.385]	-	-0.045 [0.283]	-	-0.605 [0.343]
Land-use: Fencing	-	-0.762 [0.673]	-	-	-	**1.468 [0.682]**	-	-
Land-use: Distance (linear)	-	-	-	-	-	**-0.606 [0.257]**	-	-
Land-use: Distance (quad.)	-	-	-	-	-	-0.228 [0.567]	-	-
Fencing: Distance (linear)	-	-	-	**-0.863 [0.374]**	-	-	-	-0.590 [0.362]
Fencing: Distance (quadratic)	-	-	-	-0.673 [0.766]	-	-	-	0.035 [0.546]
Land-use: Fencing: Dist(lin.)	-	-	-	-	-	-	-	-
Land-use: Fencing: Dist(quad)	-	-	-	-	-	-	-	-
VC for random effects	VC	VC	VC	VC	VC	VC	VC	VC
Site	1.36744	1.03178	0.86824	0.92847	0.99407	0.51613	1.48577	0.76802
Random edge effect	1.00423	0.77877	1.40182	1.56837	1.16948	0.76942	2.03379	1.30369
Distance block within site	0.24074	0.22113	0.31801	0.30162	0.17355	0.17130	0.12113	0.12597
Residuals	0.18424	0.18422	0.22247	0.22242	0.10841	0.10843	0.10228	0.10229
VC for fixed effects	-	0.38766	-	0.09223	-	0.48210	-	0.88316
PCV_[Site]_	-	24.55%	-	−6.94%	-	48.08%	-	48.31%
PCV_[Edge.Slope]_	-	22.45%	-	−11.88%	-	34.21%	-	35.90%
PCV_[Edge.Block]_	-	8.15%	-	5.15%	-	1.30%	-	-4.00%
$R_{GLMM(m)}^{2}$	-	14.92%	-	2.90%	-	23.53%	-	27.73%
$R_{GLMM(c)}^{2}$	-	92.91%	-	92.84%	-	94.70%	-	96.79%
AIC	618.323	601.435	681.728	679.750	492.233	468.010	475.999	462.145
**Response variable:**	**Total P**		**Total Cd**		**Total U**			
	**Null model**	**Full model**	**Null model**	**Full model**	**Null model**	**Full model**		
Fixed effects	*b* [±1 SE]	*b* [±1 SE]	*b* [±1 SE]	*b* [±1 SE]	*b* [±1 SE]	*b* [±1 SE]		
Intercept	**0.740 [0.077]**	**1.135 [0.099]**	0.113 [0.010]	**0.155 [0.009]**	**0.368 [0.054]**	**0.431 [0.062]**		
Pasture slope	-	-	-	0.018 [0.012]	-	-		
Forest slope	-	0.069 [0.103]	-	-	-	**0.231 [0.067]**		
Patch area	-	-	-	**0.050 [0.014]**	-	0.073 [0.074]		
Patch area	-	**0.275 [0.106]**	-	**0.047 [0.014]**	-	-0.057 [0.070]		
Land-use intensity gradient	-	**0.821 [0.305]**	-	**0.039 [0.014]**	-	**-0.440 [0.175]**		
Fencing	-	**-0.478 [0.132]**	-	**-0.074 [0.013]**	-	**-0.222 [0.072]**		
Distance from edge (linear)	-	**-0.365 [0.096]**	-	**-0.039 [0.012]**	-	-0.021 [0.042]		
Distance from edge (quadratic)	-	**-0.327 [0.161]**	-	**0.047 [0.021]**	-	0.080 [0.074]		
Land-use: Fencing	-	**-0.856 [0.335]**	-	-	-	**0.498 [0.176]**		
Land-use: Distance (linear)	-	**-0.947 [0.290]**	-	-	-	**-0.234 [0.085]**		
Land-use: Distance (quad.)	-	**-1.314 [0.486]**	-	-	-	0.096 [0.148]		
Fencing: Distance (linear)	-	-0.084 [0.128]	-	-	-	-		
Fencing: Distance (quadratic)	-	0.218 [0.215]	-	-	-	-		
Land-use: Fencing: Dist(lin.)	-	**1.072 [0.322]**	-	-	-	-		
Land-use: Fencing:Dist(quad)	-	**1.801 [0.539]**	-	-	-	-		
VC for random effects	VC	VC	VC	VC	VC	VC		
Site	0.15486	0.07817	0.00210	0.00061	0.06261	0.04223		
Random edge effect	0.25958	0.06250	0.00640	0.00330	0.04456	0.05459		
Distance block within site	0.03300	0.03064	0.00109	0.00109	0.01554	0.01455		
Residuals	0.02335	0.02334	0.00103	0.00103	0.00362	0.00362		
VC for fixed factors	-	0.10318	-	0.00202	-	0.04491		
PCV_[Site]_	-	49.52%	-	70.91%	-	32.55%		
PCV_[Edge.Slope]_	-	75.92%	-	48.45%	-	-22.51%		
PCV_[Edge.Block]_	-	7.14%	-	-0.37%	-	6.36%		
PCV_[Residuals]_	-	0.01%	-	-0.19%	-	0.00%		
$R_{GLMM(m)}^{2}$	-	34.64%	-	25.11%	-	28.08%		
$R_{GLMM(c)}^{2}$	-	92.16%	-	87.20%	-	97.73%		
AIC	3.206	-26.940	-1021.154	-1051.961	-490.015	-509.021		

AIC, Akaike Information Criterion; PCV, proportion change in variance; VC, variance components. For full models, the intercept represents the edge of unfenced forest remnants at low surrounding land-use intensity. Model parameters were calculated using restricted maximum likelihood (REML) estimation, and a weighted model averaging approach. Bolded coefficients are significantly different from zero (P<0.05).

**Figure 4:**
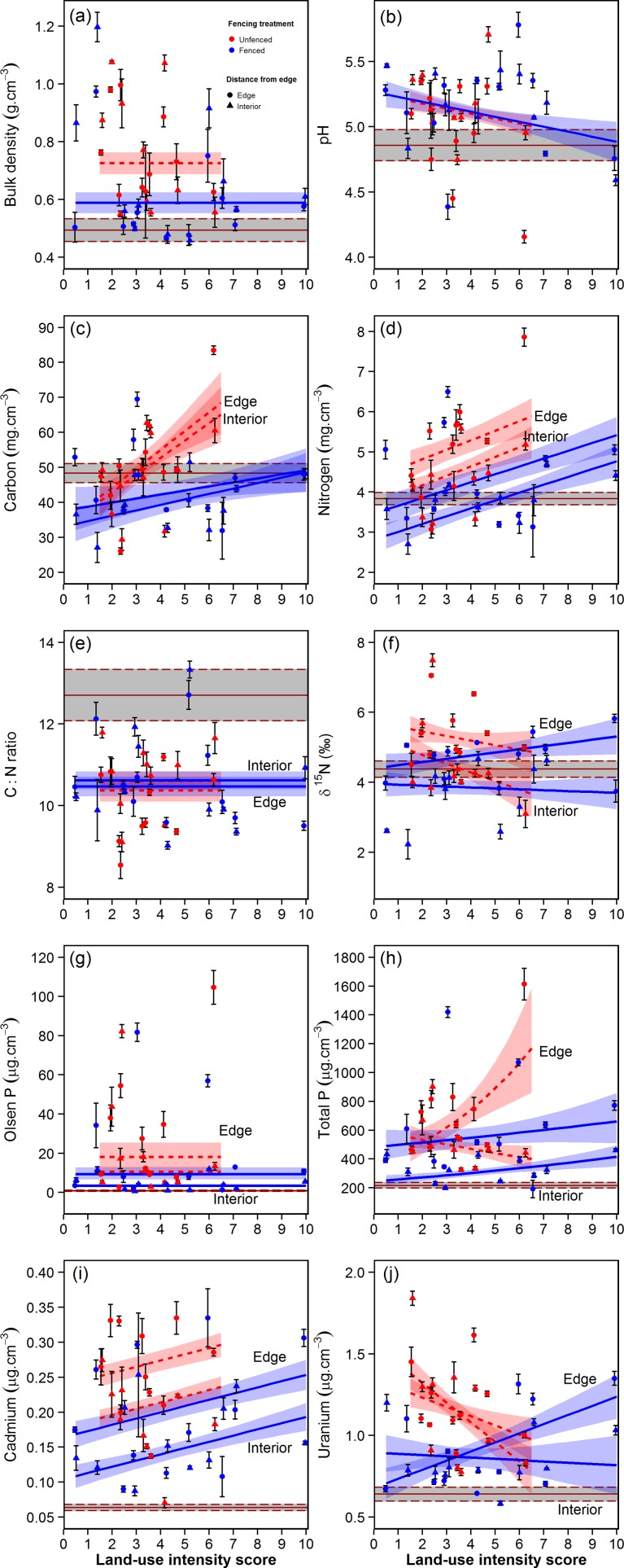
Predicted relationships between surrounding agricultural land-use intensity and soil biogeochemistry within fenced and unfenced forest remnants. The mean (± 1 S.E.) fitted relationships were derived from the final model-averaged solution (Table 2) using the ‘lmePredict’ function, while holding other fixed effects constant at their mean values. Symbols represent the raw means (± 1 S.E.), corrected for soil moisture factor and bulk density where appropriate. For clarity, raw data and predicted relationships are only shown for the extreme edge (0 m) and interior (46.5 m) distances within forest remnants, but models were tested across all edge distances. Grey shaded intervals represent the raw mean (± 1 S.E.) reference forest interior conditions (at 243–420 m from edge). Note that the predicted relationships are conditional on the random effects specified in the model.

Bulk density, on the other hand, was significantly higher in forest remnants than in forest reference sites, particularly for unfenced remnants (Table 2, Fig. 4a). There was also a significant positive effect of both adjacent pasture slope and ‘recent change in intensity’ on bulk density within forest remnants (Table 2). Interestingly, there were no significant edge-to-interior gradients in bulk density, as might have been expected for livestock trampling effects in unfenced remnants (Table 2).

After correcting for stochastic variability in soil moisture factor and spatial structuring of bulk density, we found that surrounding land-use intensity had a significant influence on seven out of the nine soil biogeochemistry variables measured (Table 2, Fig. 4), often through a complex set of interactions with fencing treatment and edge effects. For soil pH, forest remnants had significantly higher average pH than the forest reference sites, irrespective of fencing treatment (Table 2). There were also significant covariate effects of topography (with steeper forest remnants located on farms with shallower average pasture slopes tending to have lower pH values), but the dominant effect in the model was a significant decline in pH with both a higher ‘recent change in intensity’ and higher surrounding land-use intensity on farms (Table 2). Together, these fixed effects accounted for 37.17% of the site-level variance component in the model (PCV_[Site]_ in Table 2), but could not explain any significant between-site variability in edge responses (Table 2). Holding the covariate effects constant at their mean values, Fig. 4b shows the predicted decline in forest remnant pH with increasing agricultural land-use intensity on surrounding farms. However, it is important to point out that the final pH model had a relatively modest (11.75%) marginal deviance explained (i.e. for the fixed, not random, components in the model) (Table 2). Moreover, only at the highest values of land-use intensity (and at a few stochastic near-edge locations) did soil pH decline below values observed in the reference forest sites (Fig. 4b).

Soil C and N dynamics were strongly collinear, both across farmland soils (Pearson r = 0.95, n = 21, P <0.001; S1 Fig.) and across forest remnants (Fig. 4c-e). In the forest remnants, soil C was influenced by a strong three-way interaction between adjacent land-use intensity, fencing and distance from edge, after accounting for a significant positive effect of patch area on soil C (Table 2). At low land-use intensity, fenced and unfenced remnants had similar soil C values at all distances from edge (Fig. 4c), which were marginally but not significantly lower than those observed at the forest reference sites (Table 2). At higher levels of surrounding land-use intensity, soil C increased significantly, particularly in unfenced compared with fenced remnants (Fig. 4c). Similarly, soil N was significantly higher in unfenced versus fenced remnants, and increased significantly with increasing agricultural land-use intensity (Fig. 4d). However, unlike soil C there was also a significant pattern of elevated soil N at remnant edges compared with interiors, and no significant interaction effects between predictors in the model (Fig. 4d, Table 2). The collinearity of C and N responses is evident in the uniformity of the C:N ratio across forest remnants (Fig. 4e). The C:N ratio was significantly lower in forest remnants than in the interior of the reference sites (Table 2), but did not vary significantly with land-use intensity, and was only weakly influenced by an interaction between fencing and distance from edge (Table 2). Overall, the fixed predictors in the model only explained 2.90% of the deviance in the C:N ratio (Table 2).

Variation in δ^15^N enrichment across remnants (Fig. 4f) indicated more complex N dynamics than were suggested by the comparatively straightforward patterns of accumulating soil N with increasing land-use intensity (Fig. 4d). Soil δ^15^N was influenced significantly by interaction effects between land-use intensity and fencing treatment, and between land-use intensity and edge effects, after first accounting for a significant negative effect of patch area on δ^15^N enrichment (Table 2). At low land-use intensity, there was comparatively little difference in δ^15^N enrichment between the edge and interior of remnants, but significantly elevated δ^15^N values in unfenced compared with fenced remnants (Fig. 4f). By contrast, at high land-use intensity, δ^15^N values were much more strongly elevated at the edge compared to the interior of forest remnants, but there was no longer any significant difference between unfenced and fenced remnants (Fig. 4f). Overall, the fixed predictors in the model characterised variation in δ^15^N exceptionally well, explaining almost half of the site-level variance component (PCV_[Site]_ = 48.08%) and one-third of the edge-level variance component (PCV_[Edge.Slope]_ = 34.21%) (Table 2).

Soil P was dramatically elevated at almost all sites sampled within forest remnants, with available Olsen P typically ~5–30 μg.cm^-3^ in remnant interiors and ~10–100 μg.cm^-3^ at remnant edges, compared with ~2 μg.cm^-3^ at forest reference sites (Fig. 4g). Similarly, total soil P was typically ~300–600 μg.cm^-3^ in remnant interiors and ~600–1600 μg.cm^-3^ at remnant edges, compared with ~200 μg.cm^-3^ at forest reference sites (Fig. 4h). Surprisingly, there was no significant effect of surrounding land-use intensity on available Olsen P (Fig. 4g), whereas the opposite was true for total P, with complex interaction effects observed between land-use intensity and fencing treatment, and between land-use intensity and edge effects (Table 2). The final model explained almost half of site-level variance in the total P data (PCV_[Site]_ = 49.52%) and the majority of the edge-level variance component (PCV_[Edge.Slope]_ = 75.92%) (Table 2). For total P, these effects appeared to be driven predominantly by contrasting land-use effects on unfenced, compared with fenced, remnants (Fig. 4g,h). In fenced remnants, there was a comparatively shallow increase in total P with increasing land-use intensity, and the edge to interior difference in total P was similar at all levels of surrounding land-use intensity (Fig. 4g,h, Table 2). In unfenced remnants, by contrast, there was a very large influence of land-use intensity on edge to interior gradients in total P (Fig. 4h), although this relationship was partly driven by high leverage from a single unfenced edge site at high land-use intensity (Fig. 4h).

Heavy metal accumulation, as a marker of cumulative rock phosphate fertiliser addition through time, gave somewhat conflicting trends compared to standing gradients in Olsen P and total P (Fig. 4i,j). Soil cadmium levels, in particular, were dramatically elevated above baseline forest reference conditions, and showed clear and consistent evidence for elevated long-term P fertilizer application with increasing surrounding land-use intensity. This was particularly marked in unfenced compared with fenced remnants, and edges compared with interiors (Fig. 4i). Unlike total P, there were no significant interaction effects between fixed predictors in the final model-average for Cd (Table 2). The final model explained the majority of site-level variance in the Cd data (PCV_[Site]_ = 70.91%) and almost half of the within-site, edge-level variance component (PCV_[Edge.Slope]_ = 48.45%) (Table 2). Uranium levels were also highly elevated above baseline conditions (Fig. 4j), but the predicted relationships with land-use intensity were more similar to those observed for total P (Table 2). There was a significant positive effect of surrounding land-use intensity on soil U in fenced remnants, but a significant negative relationship observed in unfenced remnants (Fig. 4j). In both cases, edge-to-interior differences in soil U became greater with increasing land-use intensity (Table 2).

Finally, we repeated all the analyses without first undertaking bulk-density correction of biogeochemistry measures (i.e. for gravimetric measures of percent C, percent N, and concentrations of Olsen P, total P, Cd and U), to verify that model predictions were not unduly influenced by fencing and edge effects on bulk density (S7 Table, S8 Fig.). Both fencing and edge effects still contributed significantly to the final model-averaged predictor set in all gravimetric response models except the Olsen P model, although in several cases these had weaker statistical effects than in volumetric response models (i.e. those taking bulk-density variation into account). None of the conclusions regarding land-use intensity effects on soil biogeochemistry were qualitatively altered in the gravimetric response models (S7 Table, S8 Fig.).

## Discussion

Intensive agricultural practices can have substantial off-site influences beyond their local farm-scale application. The full implications of these effects for biodiversity conservation depend on the degree to which agricultural spillover effects scale in magnitude with increasing land-use intensification. Here, we found a strong signal of exacerbated livestock and nutrient spillover effects into native forest remnants embedded within increasingly intensive agricultural landscapes. For the majority of soil biogeochemistry measures we tested, anthropogenic influences on native forest fragments scaled in proportion to the level of land-use intensity on adjacent farms, particularly for cumulative nitrogen and phosphorus fertiliser effects. These effects were evident not just as localised small-scale edge effects at the native forest boundary, but as pervasive influences on soil properties in fragment interiors, affecting both unfenced fragments as well as fenced fragments where livestock had been excluded. Our results highlight the very real conflict that exists between strategies to intensify production land use in one part of the landscape, while at the same time trying to conserve species within adjacent natural habitats set aside for biodiversity conservation. We discuss how the scaling of these spillover effects with increasing land-use intensification might influence land sparing strategies for biodiversity conservation in landscape mosaics.

### The transition to intensive farming systems

We characterised variation in soil biogeochemistry across a continuous gradient in intensity of farming practices, from low-input:low-output agricultural systems (such as extensive sheep grazing) to high-input:high-output systems (such as intensive dairy farming) in a pastoral farming landscape in northern New Zealand. The variation in intensity across farms provided an effective space-for-time substitution for the long-term socioeconomic and agronomic transition toward intensive farm production which has occurred throughout New Zealand over the last 30 years [42, 45]. On-farm measures reflecting recent farmer inputs (N input, P input, lime input, and stocking rate) and the cumulative history of changes in soil biogeochemistry (pH, Olsen P, total C, total N, C:N ratio, δ^15^N, total P, total Cd, and total U) all increased in concert, suggesting a strong congruence between short-term and long-term indicators of intensification at most sites. The few exceptions to this general trend (sites F2, F10, U8, U9) all had higher recent farmer inputs than would have been expected based on the accumulated history of long-term soil biogeochemical change at each site, and this almost certainly reflects the continuing transition in farming systems in the region (i.e. 4 out of 21 farms, ~20%, undergoing recent intensification of agricultural practices at the time of the study). We were not able to obtain for our study system any explicit measures of primary productivity, production output, or profit, with which to calculate intensification in broader terms of farm outputs [16] or outputs-per-unit-input [88]. Nevertheless, independent evidence suggests that farm outputs and profit increase across the gradient of farming systems we studied (see farm monitoring reports at http://www.mpi.govt.nz/news-resources/publications?title=Farm%20Monitoring%20Report; accessed 08 June 2014).

The consequences of changing agricultural practices for soil properties at the farm scale have been the subject of considerable interest and concern for agronomists, public health officials, environmental scientists and policy makers (e.g., [42, 51, 56, 57, 66]). Our on-farm soil biogeochemistry data mirrored those of recent nationwide surveys of soil properties [50, 51, 54, 57, 58], in that there was a very strong signal of land-use impact on total N, δ^15^N, Olsen P, total P, total Cd and total U across farms. These land-use effects appeared to have an over-riding influence on soil properties, over and above any inherent variation that might have occurred among soil types prior to conversion to agriculture.

### Spillover from intensive farming into adjacent natural habitats

It is widely recognised that there are substantial off-site effects of farming practices that spill over into adjacent aquatic ecosystems [89–91], and policies have frequently been adopted to mitigate these effects [92]. In contrast, there have been few comparable attempts to monitor, or mitigate, potential spillover effects into adjacent terrestrial ecosystems beyond the farm boundary [29]. Here, we found that adjacent land-use intensity was an important driver of changes in soil biogeochemistry within forest remnants, even after accounting for significant covariate effects of farmland slope, forest slope, and remnant patch area. We briefly discuss these spillover effects in relation to the key effects of land-use intensification on pH, soil C content, N-cycling dynamics and P-dynamics in adjacent forest fragments. However, a detailed analysis of each specific spillover effect is unwarranted here as it is the combined effect of multiple and interacting transfers into adjacent ecosystems that is likely to have the greatest impact on ecosystem function rather than individual transfers.

The surface soil pH of forest fragments was significantly elevated above the moderately-acidic levels (pH 4.8) typical of native forest conditions in the region. The pH of fragments appears to be tending towards the adjacent agricultural soil pH conditions that farmers in the region attempt to achieve for optimal pasture growth (pH 5.5) using lime addition [93, 94]. If this is the case, then it would suggest that lime spillover from adjacent agriculture (via drift from aerial dispersal or run-off) is ubiquitous across the landscape, influencing soil pH conditions throughout both the interior and the edges of all small forest fragments in the region. The data indicate that elevated pH levels are more pronounced in relatively flat forest fragments on otherwise relatively steep terrain, suggesting a significant run-off component to the spillover of lime. The consequences of elevated soil pH for ecosystem processes in native forest fragments will be to alter availability of some critical nutrients, such as phosphorus [78], and potentially decrease aluminium and manganese toxicity [94]. Importantly, the high spillover effect on pH was not exacerbated by increasing land-use intensification on surrounding farms, most likely because farms aim for a soil pH optima of between 5 and 6 irrespective of land use intensity.

Anthropogenic N addition, through N fertiliser inputs or clover N fixation, had a large influence on N spillover into forest fragments, with soil total N being significantly elevated at forest edges in particular, but also in fragment interiors. These effects were exacerbated in fragments surrounded by farms with higher land-use intensity. Similarly, soil C content in forest fragments increased significantly with increasing agricultural land-use intensity on surrounding farms. The causal mechanism driving this relationship is not yet clear, as spatial variation in total C is typically explained more by underlying soil type than by land use [57]. Broad-scale comparisons of New Zealand topsoils have typically shown that indigenous forest and long-term pasture have similar total C contents [50]. Whether our findings suggest a causal link between intensive farming activities and an increase in total C content in adjacent forest fragments remains to be tested (experimentally). However, the strength of N addition effects on soil properties was evident in the strong covariance between soil N and soil C contents (both across farms and across edge gradients within fragments), such that C:N ratios across sites were almost invariant at very low levels (~9–10). This is strikingly different from typical values observed across different land-use types in New Zealand, with a broad-scale survey [58] reporting that the C:N ratio for native forest sites (n = 58) was typically ~16 (more similar to our reference sites at ~13), while the C:N ratio for dairy farming sites (n = 127) was typically ~11. In the present study, the average C:N ratio for forest fragments was dramatically lower than at reference forest locations, and was as low as values observed for many farmland soils [58]. Moreover, the observed C:N values are close to the lower limit possible for soils, determined by covalent binding of N with organic matter [95]. In broad terms, then, this suggests that the majority of our fragment soils have accumulated substantial N, and it is this N saturation which is driving the covariance in soil N and C relationships. Given the high N contents and low C:N ratios of the forest fragment soils, it would have been expected that changes in δ^15^N would also respond strongly to increasing land-use intensity. Average δ^15^N values were generally greater than those reported for forests (2.1‰) and closer to typical values for sheep/beef farms (3.8 ‰) and dairy pastures (5.4 ‰) [51]. This supports the suggestion that topsoils in all the fragments have accumulated substantial N and experience increased N cycling (as also indicated by generally low C:N ratios).

Soil phosphorus dynamics were significantly altered in forest fragments compared to reference forest sites. There was clear evidence of transfer of applied P fertiliser from farmland into adjacent forest remnants, with concentrations of Olsen P, total P, U and Cd being dramatically elevated in all fragments. The relative weight of evidence from the four markers of phosphatic fertilizer accumulation was varied (presumably because of different soil binding properties and soil residence times), so we focus on the cumulative evidence for changes in soil phosphorus dynamics. Importantly, all variables showed evidence for substantial P fertilizer transfer and P accumulation at all distances from the forest edge to the centre of each patch (typically at levels two- to five-fold greater than those observed in reference forest interiors). There was also evidence that fencing had a large influence on transfer rates, with greater P and heavy-metal contaminants accumulating in both the edge and centre of fragments that were not fenced. This suggests that P transfer in animal excreta is an important component of phosphorus dynamics in forest remnants, over and above P spillover effects from run-off and aerial drift.

### Conservation protection for forest fragments embedded in production landscapes

Across all soil biogeochemistry measures, spillover effects were typically more severe at the edge compared with the interior of forest fragments, and were exacerbated in fragments with open livestock access. To some extent, then, fencing to exclude livestock-driven soil disturbance and nutrient inputs can be seen as the first crucial step in mitigating the most extreme spillover effects. However, even in the interior of fenced fragments (approximately 50 m from the edge), soil properties were still significantly different from values observed in the interior of reference sites (up to 420 m from the edge). This is in line with observed changes in a range of other ecosystem properties, such as vegetation structure and microclimate, within 50 m of the forest edge [96–98]. Moreover, spillover rates were exacerbated even further at high levels of surrounding land-use intensity, despite fencing protection. This tends to suggest that a substantial component of land-use impact occurs via nutrient spillover channels (e.g., run-off and aerial drift), not just dung and urine addition associated with livestock spillover. Consequently, the effective management of such pervasive agricultural spillover effects will require either reduction in management intensity or active mitigation of flow rates at the source (on-farm), or the creation of buffer interception zones between managed and natural habitats (in the wider landscape). For example, riparian buffer zones are widely promoted as a management tool for mitigating spillover from farmland into adjacent aquatic systems, because they prevent sedimentation and polluted runoff from reaching waterways and are effective at improving water quality and reducing pollution [99, 100]. Comparable examples of vegetated buffer zones being used to mitigate spillover between managed and natural terrestrial systems are rare [30, 101]. Despite this, similar principles can almost certainly be applied, such as planting a combination of vegetation types to intercept a wide range of nutrient flows (e.g., grass buffers are more effective for nutrient transport in sediments, and forested buffers for soluble nutrient transport; [100, 102, 103]). Additionally, vegetated buffer zones surrounding forest fragments will not only provide mitigation of nutrient spillover but potentially provide other benefits to the forest ecosystem such as reduced microclimatic edge effects on plant and animal communities [104].

A failure to consider agricultural spillover effects is likely to have severe consequences for biodiversity within landscapes dominated by intensive agriculture. This is particularly relevant for small habitat remnants embedded within production landscapes [34, 48, 61], which now dominate most lowland agricultural regions of the world (e.g., [59, 105]). Nutrient enrichment of soils, such as we observed with increasing adjacent land-use intensity, can have substantial impacts on above- and below-ground biodiversity and ecosystem functioning. Increasing ecosystem productivity can reduce plant species richness [106] and alter species composition [107, 108], including in woodlots adjacent to high-intensity farms [30]. Such changes in composition can result from the increased susceptibility of communities to invasion, both by species that specialise on specific resources (e.g. nitrophilic species, [107]) and generally due to the increased niche availability associated with resource addition [109]. In addition to the consequences of altered plant biodiversity for various measures of ecosystem functioning [110], changes to the amount and spatial distribution of soil resources can moderate the relationship between plant diversity and ecosystem functioning [108, 111, 112], and alter the diversity and mean value of functionally-important plant traits [108]. Changes to plant species composition and biomass can also drive significant changes at higher trophic levels, for example by altering herbivore phenology and biomass, both in absolute terms and relative to other trophic levels [113, 114]. Moreover, interspecific differences in the biomass changes of herbivores can alter their attractiveness to natural enemies, and thereby drive changes in attack rates and food-web structure [115].

### The future of land-sparing options in mosaic landscapes

Our findings challenge the general utility of intensification strategies to provide optimal solutions for crop yield versus biodiversity trade-offs [3, 8], or livestock production versus greenhouse gas emission trade-offs [116], without appropriately considering spillover effects at local and landscape scales. The apparent benefits of increased yield must be explicitly weighed against the increasing magnitude of livestock and nutrient spillover from intensive agriculture into adjacent natural habitat. Such spillover effects are likely to be most problematic in fine-grained production mosaics, where natural habitats are highly fragmented and interspersed among intensive agricultural areas (as in this study), and where lateral flow rates of nutrients, livestock and other organisms are high. Of course, there is likely to be an additional suite of negative externalities that accrue across larger (regional or global) scales due to local intensification of agricultural practices, but our results do not address key global governance issues stemming from globally interconnected food systems [36].

Of some cause for concern at landscape scales is the large number of soil properties in forest fragments (particularly bulk density, pH, Olsen P, total P and total Cd) which were significantly affected by an apparent recent change in intensity within the surrounding agricultural matrix. This suggests that spillover effects can be exacerbated over relatively short time intervals, even for some cumulative measures of soil biogeochemistry such as total P and Cd, perhaps where phosphatic fertilizer application rates are very high following conversion from low- to high-intensity farming systems [117].

### Conclusions

An increasing number of studies are showing that historical land-use can have long-lasting (potentially irreversible) effects on current and future soil processes (e.g. [118, 119]). This suggests that we should be very concerned about the cumulative effects of nutrient and livestock spillover into forest remnants within agricultural mosaics. Small forest remnants, in the size range of 2–16 ha studied here, frequently represent the only remaining natural habitats in lowland agricultural landscapes [34, 59, 105], and we found strong evidence for the pervasive alteration of soil properties in these systems. The consequences of increased soil compaction, increased pH, dramatically decreased C:N ratio, N saturation, and a two- to five-fold increase in P loading and heavy-metal contamination are likely to be severe for biodiversity and ecosystem functioning within forest fragments, because soil nutrient availability can affect biodiversity, species traits, and various measures of ecosystem functioning [108]. Below-ground versus above-ground linkages are recognised as being critical for structuring ecological communities and regulating ecosystem processes [49], but much more emphasis needs to be placed on modelling the interactions and negative feedback effects between land-use intensification, spillover, and altered landscape processes [120, 121] in agricultural landscape mosaics.

## Supporting Information

S1 Supporting Information Quantifying a land-use intensity gradient across farms. (DOCX)

S1 Table A representative stock unit conversion table for the Waikato region (http://www.waikatoregion.govt.nz/Environment/Environmental-information/Environmental-indicators/Land-and-soil/Land/riv9-technical-information/; accessed 19 June 2012). (DOCX)

S2 Table Breakdown of stocking rates (SU: stock units) by livestock class for farms surrounding the 21 forest remnants. Site codes and superscript symbols as in Table 1. An asterisk (*) indicates no information available on breakdown by livestock class.
(DOCX)

S3 Table New Zealand Soil Classification (NZSC) codes for the paddocks surrounding the 21 forest remnants and three forest reference sites, detailing a range of relevant characteristics. (DOCX)

S4 Table Covariate effects tested in the mixed effects models for the influence of agricultural land-use intensity on farms surrounding the 21 forest remnants. ‘F’ and ‘U’ site codes indicate whether the adjacent forest remnants were fenced or unfenced.
(DOCX)

S5 Table Eigenvectors for the factor correlation matrix in the PCA ordination. The eigenvectors give the coefficients for the linear combination of variables which defines the PCA axis.
(DOCX)

S6 Table Components of variation in agricultural land-use intensity on farms surrounding the three forest reference sites, Maungakawa Reserve (MKRes; 965 ha), Te Miro Scenic Reserve (TMRes; 403 ha) and Te Tapui Reserve (TTRes; 1377 ha). Values for ‘Farm area’ only represent the farm adjacent to the sampling sites, but larger forest reserves are surrounding by many other farms as well. All soil measures are the average of three recorded values taken in open pasture 46.5 m outside the forest edge at each site.
(DOCX)

S7 Table Results of mixed-effects modelling for gravimetric measures of soil nutrient geochemistry in native forest remnants embedded within production landscapes. (DOCX)

S1 Fig Correlation matrix for the 13 measures of agricultural land-use intensity on farms surrounding the 21 forest remnants. Variables were transformed where necessary (see text) and normalised for subsequent PCA ordination analysis. Values above the diagonal are Pearson correlation coefficients, size-scaled by the value of the coefficient.
(PDF)

S2 Fig Factor loadings (correlations) on PCA axes 1 and 2 showing the dominant farmer input measures and soil biogeochemistry measures driving variation in land-use intensity across farms. (PDF)

S3 Fig Sensitivity test of the influence of using short-term versus longer-term land-use intensity measures to derive a composite overall land-use intensity gradient. See text for explanation.
(PDF)

S4 Fig The biplot of PCA axes 1 and 2 from Fig. 2 (based on all 13 farmer input and soil biogeochemistry measures) re-plotted to show variation in soil groups (symbols: BO = Typic Orthic Brown Soils; LO = Typic Orthic Allophanic Soils; RO = Mottled Orthic Recent Soils) and names of soil types for each farm. No consistent clustering or confounding effect of soil type was evident along the PCA axis 1 land-use intensity gradient.
(PDF)

S5 Fig Spline correlogram showing whether the spatial location of sampling sites in the landscape had a significant effect on the pairwise similarity of PCA axis 1 land-use intensity scores among sites. The mean (± 95 confidence limits) for Moran’s I indicate whether the observed pairwise similarity at a given scale is significantly greater or less than expected by chance alone, using a resampling procedure with 1000 random draws in the ncf package in R2.14.2.
(PDF)

S6 Fig Comparative analysis of where farms adjacent to the three forest reference sites (blue circles) were placed along the composite land-use intensity gradient for farms surrounding the 21 forest remnant sites (red triangles). See text for further explanation.
(PDF)

S7 Fig Predicted relationships between surrounding agricultural land-use intensity and soil moisture factor within fenced and unfenced forest remnants. The mean (± 1 S.E.) fitted relationships were derived from the final model-averaged solution (Table 2) using the ‘lmePredict’ function, while holding other fixed effects constant at their mean values. Symbols represent the raw means (± 1 S.E.). The grey shaded interval represents the raw mean (± 1 S.E.) of the soil moisture factor at the reference forest interior sites (243–420 m from edge). Note that the predicted relationships are conditional on the random effects specified in the model.
(PDF)

S8 Fig Predicted relationships between surrounding agricultural land-use intensity and soil biogeochemistry within fenced and unfenced forest remnants, replotted as gravimetric response measures rather than as volumetric response measures. See Fig. 4 for details.
(PDF)
