# Supplementary material for: Agricultural Intensification Exacerbates Spillover Effects on Soil Biogeochemistry in Adjacent Forest Remnants
Source: PLoS One. 2015 Jan 9;10(1):e0116474. doi: 10.1371/journal.pone.0116474 (PMC4289067; doi:10.1371/journal.pone.0116474)
Supplement: S1 Supporting Information — (DOCX) [file pone.0116474.s001.docx]

**Supporting Information S1**

### Quantifying a land-use intensity gradient across farms

***Principal Components Analysis***

We had no a priori evidence for a single variable that would represent an ‘ideal’ indicator of land-use intensity across farms. Instead, it was highly likely that different variables would vary in their influences on soil biogeochemistry responses within forest remnants. However, the relative weighting of these influences could not be predicted in advance. Moreover, the 13 measures of farmer inputs and soil biogeochemistry showed varying degrees of collinearity across sites, including strong correlations between short-term and longer-term variables (Figure S1). Consequently, we chose to construct an unweighted composite index of land-use intensity across farms using all available measures. The most appropriate technique for distilling these types of multivariate environmental data into a composite gradient of land-use intensity is a correlation-based Principle Components Analysis (PCA) (Clarke and Warwick 2001, Clarke and Gorley 2006). Resemblance among samples of environmental data is considered to be best described by the Euclidean distance metric in a PCA ordination, because there is a mix of measurement scales across variables, negative values can occur, zeros have no special meaning, and positive similarity can always be inferred for two samples that have the same value, even if that value is zero (Clarke and Warwick 2001, Clarke and Gorley 2006).

***High correlation of short-term versus long-term measures of land-use intensity***

The relevant time-scales of influence of different land-use measures were not precisely known. However, our *a priori* expectation was that the four farmer-input measures calculated for the last 5 yrs (N input, P input, lime input, and stocking rate) as well as two labile soil biogeochemistry measures (pH and Olsen P) would reflect comparatively short-term land-use change, while the remaining seven soil biogeochemistry measures (total C, total N, C:N ratio, δ^15^N, total P, total Cd, and total U) would reflect longer-term historical land-use impacts. We had no objective means by which to differentially weight individual variables in constructing a composite land-use intensity gradient. Although we noted in the overall PCA ordination that there were highly significant positive correlations between many of these recent versus historical land-use measures (Figures S1, S3, S4), there were outlier sites (Figure 2) that appeared to show a mis-match between evidence of land-intensity based on short-term versus long-term measures. Consequently, we conducted a sensitivity test of the degree of consensus between composite rankings of land-use intensity based on short-term versus longer-term variables.

In the sensitivity test we conducted two separate PCA ordinations, with one based on the six ‘recent’ land-use measures (PCA axes 1 and 2 explained 66.7% of variation in all short-term measures combined), and one based on the seven ‘historical’ land-use measures (PCA axes 1 and 2 explained 67.5% of variation in all longer-term measures combined). We correlated relative site-to-site dissimilarity in ordination space between the two approaches, and found a significant positive correlation between recent versus historical land-use intensity based on PCA axis 1 scores (Pearson's correlation, r = 0.49, p = 0.025; Figure S3a). Similarly, we tested the degree of correlation based on the full Euclidean dissimilarity matrix using the ‘RELATE’ function in Primer v6 (all five PCA axes at once) and there was a significant rank correlation of relative land-use intensity scores between recent versus historical land-use measures (Spearman’s Rho=0.231, P=0.041; 9999 random permutations).

The outlier sites identified in Figure S3a (sites below the diagonal line) were the same outlier sites identified in the composite land-use intensity gradient in Figure 2, confirming that there is a mis-match in evidence based on short-term versus long-term measures in these cases. Sites that fall well below the ‘y=x’ line in Figure S3a are sites that rank much more highly for short-term inputs than they do for historical soil geochemistry measures, relative to other sites. We interpret this as cases where farmers have recently increased the intensity of land-use practices in these paddocks, which otherwise have a soil legacy of relatively low land-use intensity.

Consequently, we calculated the residuals around the recent-vs-historical land-use relationship in Figure S3a as an index of ‘recent change in intensity’, scaled so that high values indicate high recent increase in land-use intensity (Figure S3b). We confirmed that there was no significant correlation between ‘recent change in intensity’ and the original composite index of land-use intensity based on all 13 farmer input and soil biogeochemistry measures (Pearson's correlation r = -0.25, p = 0.274; Figure S3b); i.e., ‘recent intensification’ does not co-vary with absolute intensity across farms.

Finally, we tested whether correcting for ‘recent change in intensity’ had any substantial effect on the rank-ordering of sites along the composite PCA axis 1 land-use intensity gradient. To do this, we regressed the PCA axis 1 scores (based on all 13 measures) against the index of recent change in intensity, and compared the ‘adjusted’ rank ordering of the residual scores against the original rank ordering of land-use intensity across sites (Figure S3c). In this analysis, we found that the rank order of sites was essentially identical at high land-use intensity values, but rank ordering was more affected by recent change in farm intensity at lower land-use intensity values (Figure S3c). Importantly, there was no directional bias in the effects of a recent change in intensity, and we conclude from the sensitivity test that the overall PCA axis 1 land-use intensity gradient based on all 13 land-use measures is a robust, general measure of agricultural intensity across farms.

***No confounding effect of variation in soil type classification across sites***

Due to the wide geographic spread of study sites, and the non-random distribution of farming activities in the landscape, it was not possible to constrain all farms to a single soil type. The study sites spanned three soil orders/groups in the NZ Soil Classification (NZSC) scheme, split predominantly between Typic Orthic Brown Soils (12 sites) and Typic Orthic Allophanic Soils (6 sites), with fewer sites having Mottled Orthic Recent Soils (3 sites) (Table S3). Different soil types are known to have different nutrient retention properties, drainage, pH buffering capacity and susceptibility to land-use impacts (Brady and Weil 2008). Therefore, we quantitatively assessed the potential confounding effects of soil type on the interpretation of the land-use intensity gradient.

We plotted the distribution of soil types across sites in the composite PCA gradient of land-use intensity (Figure S4). There was no evidence of covariance between soil type and site dissimilarity in ordination space, with Pukerata silt loam hill soils distributed widely across farms with all levels of land-use intensity, and other soil types apparently scattered at random (Kruskal-Wallis rank sum test for differences in PCA axis 1 scores between the three soil groups: χ^2^ = 1.537, d.f. = 2, P = 0.464; and for differences in PCA axis 1 scores between the six soil families: χ^2^ = 3.154, d.f. = 5, P = 0.676).

As P retention is known to differ strongly between soil orders/groups, we also conducted a specific test of whether P availability in paddocks co-varied with soil group, but there was no significant effect (Kruskal-Wallis rank sum test for differences in Olsen P between the three soil groups: χ^2^ = 0.234, d.f. = 2, P = 0.890; or between the six soil families: χ^2^ = 5.848, d.f. = 5, P = 0.321).

Similarly, as pH buffering capacity is known to differ strongly between soil orders/groups, we conducted a specific test of whether farmer lime input co-varied with soil group, and there was a small but significant effect (Kruskal-Wallis rank sum test for differences in recent lime input between the three soil groups: χ^2^ = 6.382, d.f. = 2, P = 0.041; and between the six soil families: χ^2^ = 10.163, d.f. = 5, P = 0.071). Farmers at the three sites with Mottled Orthic Recent Soils tended to apply more lime, whereas farmers on Orthic Allophanic soils applied particularly low amounts of lime in the last five years. However, this recent history of farmer lime input did not seem to influence covariance between soil type and soil pH across paddocks (Kruskal-Wallis rank sum test for differences in soil pH between the three soil groups: χ^2^ = 2.037, d.f. = 2, P = 0.361; or between the six soil families: χ^2^ = 5.291, d.f. = 5, P = 0.381).

Finally, we tested for evidence of covariance between soil type and the calculated ‘index of recent change in intensity’ (Figure S3b), but found none (Kruskal-Wallis rank sum test for differences in the index of recent change in intensity between the three soil groups: χ^2^ = 1.649, d.f. = 2, P = 0.438; or between the six soil families: χ^2^ = 4.527, d.f. = 5, P = 0.476).

From these analyses we conclude that our gradient of land-use intensity is not influenced or confounded by variation in soil type among sites. This suggests that the agricultural intensification of land use has had an over-riding effect on soil biogeochemistry that is greater than any inherent differences in soil type.

***No spatial autocorrelation of land-use intensity across farms***

In several instances, more than one paddock was sampled on the same farm, although we always maintained a minimum distance of 420 m between sites. As we used a mixture of farm-scale and paddock-scale variables in the PCA analysis, there is the potential for confounding spatial autocorrelation of site similarity in ordination space. In the PCA ordination (Figure 2) there was little evidence that sites located on the same landowner’s farm (see Table 1) had similar overall measures of land-use intensity (i.e., were clustered together in ordination space). To confirm this, we conducted a test of spatial autocorrelation of PCA axis 1 land-use intensity values using the spline.correlog function in the ‘ncf’ package in R 2.14.2 (Bjornstad and Falck 2001).

In the spline correlogram (Figure S5), there was no evidence that paddocks that were closer together in space (e.g., on the same farm, or adjacent farms) had PCA axis 1 values (i.e., land-use intensity scores) that were significantly more similar than expected by chance alone.

***What is the land-use intensity surrounding the forest reference sites?***

Soil biogeochemistry was measured along edge-to-interior gradients into three forest reserves (Te Miro Scenic Reserve (403 ha), Maungakawa Reserve (965 ha) and Te Tapui Reserve (1377 ha)) as reference sites against which to compare the forest remnants. In order to minimise the likely historical spillover effects of agricultural land-use into the forest reference sites, we selected the edges of forest reference sites that were up-slope from adjacent paddocks. We also selected areas adjacent to farms that appeared, *a priori*, to be of average land-use intensity (i.e., predominantly beef-dominated farming types, rather than extensive sheep farms or intensive dairy farms). To test how the degree of land-use intensity on farms surrounding the forest reference sites compared with land-use intensity on farms surrounding the forest remnants, we collected an identical series of farmer input and soil biogeochemistry measures for paddocks surrounding forest reference sites, using the methods described above (Table S6). The soil classification at all reference sites was Typic Orthic Brown Soils [BO], with Kakepuku hill soil at TTRes and MKRes, but Pukerata silt loam hill soil at TMRes (see soil characteristics for these soil types in Table S3).

Using the same procedures as described above, we conducted a PCA ordination analysis of the 13 farmer input and soil biogeochemistry measures for paddocks surrounding the three reference sites and paddocks surrounding the 21 forest remnants sites, combined. We compared site dissimilarity in ordination space with site dissimilarity in the original PCA that was conducted without the reference sites, in order to visualise the relative placement of these farms along the land-use intensity gradient. The comparative analysis (Figure S6) indicated that land-use intensity surrounding the three reference sites was typical of average land-use intensity values on farms surrounding the 21 forest remnants, with none of the farms adjacent to reference sites having high leverage in the analysis (Figure S6).

**Supplementary references**

Bjornstad, O. N. & Falck, W. 2001. Nonparametric spatial covariance functions: Estimation and testing. Environmental and Ecological Statistics 8, 53-70.

Clarke, K. R. and Gorley, R. N. 2006. PRIMER v6: User Manual/Tutorial. PRIMER-E Ltd, Plymouth U.K.

Clarke, K. R. and Warwick, R. M. 2001. Change in Marine Communities. 2^nd^ Edition. PRIMER-E Ltd, Plymouth U.K.
