# Supplementary material for: Agricultural Intensification Exacerbates Spillover Effects on Soil Biogeochemistry in Adjacent Forest Remnants
Source: PLoS One. 2015 Jan 9;10(1):e0116474. doi: 10.1371/journal.pone.0116474 (PMC4289067; doi:10.1371/journal.pone.0116474)
Supplement: S1 Table — (DOCX) [file pone.0116474.s002.docx]

**Table S1.** A representative stock unit conversion table for the Waikato region (<http://www.waikatoregion.govt.nz/Environment/Environmental-information/Environmental-indicators/Land-and-soil/Land/riv9-technical-information/>; accessed 19 June 2012).

| **Farm Type** | **Class** | **Stock Units (SU) or ewe equivalent** | **Percent of each stock ‘typically’ found on farms (%)** |
| --- | --- | --- | --- |
| Dairy | Dairy Cows | 7.00 | 73.1 |
|  | Dairy Replacements | 4.25 | 24.5 |
|  | Other (bulls etc) | 5.50 | 2.4 |
| Beef | Beef Cows | 5.50 | 21.5 |
|  | Beef Dry | 4.75 | 57.2 |
|  | Beef Replacements | 4.00 | 14.4 |
|  | Other | 5.50 | 6.9 |
| Sheep | Breeding Ewes | 1.00 | 67.6 |
|  | Sheep Dry | 0.80 | 13.8 |
|  | Sheep replacements | 0.70 | 5.9 |
|  | Other | 0.80 | 12.7 |
| Deer | Hinds | 1.90 | 49.9 |
|  | Deer for Meat | 1.80 | 30.2 |
|  | Stags for velvet | 2.10 | 5.3 |
|  | Other | 1.80 | 14.6 |
