# Supplementary material for: Agricultural Intensification Exacerbates Spillover Effects on Soil Biogeochemistry in Adjacent Forest Remnants
Source: PLoS One. 2015 Jan 9;10(1):e0116474. doi: 10.1371/journal.pone.0116474 (PMC4289067; doi:10.1371/journal.pone.0116474)
Supplement: S2 Table — Site codes and superscript symbols as in Table 1. An asterisk (*) indicates no information available on breakdown by livestock class. (DOCX) [file pone.0116474.s003.docx]

**Table S2.** Breakdown of stocking rates (SU: stock units) by livestock class for farms surrounding the 21 forest remnants. Site codes and superscript symbols as in Table 1. An asterisk (*) indicates no information available on breakdown by livestock class.

| **Site code** | **Farm area** ha | **Farming type** | **Dairy milking cows** SU.ha^-1^ | **Dairy grazing replacement** SU.ha^-1^ | **Bulls** SU.ha^-1^ | **Beef** SU.ha^-1^ | **Deer** SU.ha^-1^ | **Sheep** SU.ha^-1^ | **Total stocking rate** SU.ha^-1^ |
| --- | --- | --- | --- | --- | --- | --- | --- | --- | --- |
| F1 | 120 | Dairy | 18.8 |  |  |  |  |  | 18.8 |
| F2 | 150 | Dairy | 18.9 |  |  |  |  |  | 18.9 |
| F3 | 560 ^a^ | Beef + Dairy grazing |  | 3.6 |  | 8.4 |  |  | 12.0 |
| F4 | 270 ^e^ | Bulls |  |  | 12.2 |  | 2.1 |  | 14.3 |
| F5 | 270 ^e^ | Bulls |  |  | 12.2 |  | 2.1 |  | 14.3 |
| F6 | 120 | Dairy grazing |  | 10.6 |  |  |  |  | 10.6 |
| F7 | 404 ^d^ | Sheep + Dairy grazing |  | 7.9 |  |  |  | 4.6 | 12.5 |
| F8 | 330 | Sheep + Beef |  |  |  | 10.2 |  | 4.4 | 14.6 |
| F9 | 121 | Beef + Dairy grazing |  | 9.0 |  | 1.0 |  |  | 10.0 |
| F10 | 404 ^d^ | Sheep + Dairy grazing |  | 7.9 |  |  |  | 4.6 | 12.5 |
| F11 | 31 | Dairy grazing |  | 6.9 |  |  |  |  | 6.9 |
| U1 | 200 ^c^ | Sheep + Beef |  |  |  | 12 |  | 5.5 | 17.5 |
| U2 | 404 ^b^ | Beef + Dairy grazing |  | * |  | * |  |  | 12.4 |
| U3 | 404 ^b^ | Beef + Dairy grazing |  | * |  | * |  |  | 12.4 |
| U4 | 560 ^a^ | Beef + Dairy grazing |  | 3.6 |  | 8.4 |  |  | 12.0 |
| U5 | 275 | Dairy grazing |  | 11.3 |  |  |  |  | 11.3 |
| U6 | 200 ^c^ | Sheep + Beef |  |  |  | 12 |  | 5.5 | 17.5 |
| U7 | 180 | Sheep + Beef |  |  |  | 5.6 |  | 4 | 9.6 |
| U8 | 120 | Sheep + Dairy grazing |  | 8 |  |  |  | 8 | 16.0 |
| U9 | 192 | Sheep + Beef |  |  |  | 9.2 |  | 6.4 | 15.6 |
| U10 | 357 | Sheep + Beef |  |  |  | 7.8 |  | 3.8 | 11.1 |
