# Supplementary material for: Agricultural Intensification Exacerbates Spillover Effects on Soil Biogeochemistry in Adjacent Forest Remnants
Source: PLoS One. 2015 Jan 9;10(1):e0116474. doi: 10.1371/journal.pone.0116474 (PMC4289067; doi:10.1371/journal.pone.0116474)
Supplement: S3 Table — (DOCX) [file pone.0116474.s004.docx]

**Table S3.** New Zealand Soil Classification (NZSC) codes for the paddocks surrounding the 21 forest remnants and three forest reference sites, detailing a range of relevant characteristics.

| **Site code** | **Soil order/ group** | **Soil classification** | **Soil family** | **Soil name** | **Texture** | **Parent rock** | **Topsoil clay range** | **P retention** |
| --- | --- | --- | --- | --- | --- | --- | --- | --- |
| F1 | BO | Typic Orthic Brown Soils | Teng_16.1 | Pukerata silt loam hill soil | Loamy | Sandstone and Rhyolitic rock | 20-25% | moderate |
| F2 | LO | Typic Orthic Allophanic Soils | Ngak_13.1 | Tirau sandy loam | Loamy | Tephra | 15-20% | very high |
| F3 | LO | Typic Orthic Allophanic Soils | Otor_1.4 | Peria soils, rolling phase | Loamy | Tephra | 20-25% | very high |
| F4 | BO | Typic Orthic Brown Soils | Teng_16.1 | Pukerata silt loam hill soil | Loamy | Sandstone and Rhyolitic rock | 20-25% | moderate |
| F5 | BO | Typic Orthic Brown Soils | Teng_16.1 | Pukerata silt loam hill soil | Loamy | Sandstone and Rhyolitic rock | 20-25% | moderate |
| F6 | LO | Typic Orthic Allophanic Soils | Waite_2.2 | Otorohanga silt loam | Loamy | Tephra | 20-25% | very high |
| F7 | BO | Typic Orthic Brown Soils | Teng_16.1 | Pukerata silt loam hill soil | Loamy | Sandstone and Rhyolitic rock | 20-25% | moderate |
| F8 | BO | Typic Orthic Brown Soils | Kake_1.1 | Kakepuku hill soils | Clayey | Tephra | 20-25% | moderate |
| F9 | RO | Mottled Orthic Recent Soils | Tauw_1.1 | Tauwhare sandy loam hill soil | Loamy | Rhyolitic rock | 10-15% | low |
| F10 | BO | Typic Orthic Brown Soils | Teng_16.1 | Pukerata silt loam hill soil | Loamy | Sandstone and Rhyolitic rock | 20-25% | moderate |
| F11 | BO | Typic Orthic Brown Soils | Teng_16.1 | Pukerata silt loam hill soil | Loamy | Sandstone and Rhyolitic rock | 20-25% | moderate |
| U1 | BO | Typic Orthic Brown Soils | Teng_16.1 | Pukerata silt loam hill soil | Loamy | Sandstone and Rhyolitic rock | 20-25% | moderate |
| U2 | RO | Mottled Orthic Recent Soils | Tauw_1.1 | Tauwhare sandy loam hill soil | Loamy | Rhyolitic rock | 10-15% | low |
| U3 | LO | Typic Orthic Allophanic Soils | Waite_2.2 | Otorohanga silt loam | Loamy | Tephra | 20-25% | very high |
| U4 | LO | Typic Orthic Allophanic Soils | Otor_1.4 | Peria soils, rolling phase | Loamy | Tephra | 20-25% | very high |
| U5 | BO | Typic Orthic Brown Soils | Teng_16.1 | Pukerata silt loam hill soil | Loamy | Sandstone and Rhyolitic rock | 20-25% | moderate |
| U6 | BO | Typic Orthic Brown Soils | Teng_16.1 | Pukerata silt loam hill soil | Loamy | Sandstone and Rhyolitic rock | 20-25% | moderate |
| U7 | BO | Typic Orthic Brown Soils | Teng_16.1 | Pukerata silt loam hill soil | Loamy | Sandstone and Rhyolitic rock | 20-25% | moderate |
| U8 | BO | Typic Orthic Brown Soils | Teng_16.1 | Pukerata silt loam hill soil | Loamy | Sandstone and Rhyolitic rock | 20-25% | moderate |
| U9 | RO | Mottled Orthic Recent Soils | Tauw_1.1 | Tauwhare sandy loam hill soil | Loamy | Rhyolitic rock | 10-15% | low |
| U10 | LO | Typic Orthic Allophanic Soils | Otor_1.4 | Peria soils, rolling phase | Loamy | Tephra | 20-25% | very high |
| MKRes | BO | Typic Orthic Brown Soils | Kake_1.1 | Kakepuku hill soils | Clayey | Tephra | 20-25% | moderate |
| TMRes | BO | Typic Orthic Brown Soils | Teng_16.1 | Pukerata silt loam hill soil | Loamy | Sandstone and Rhyolitic rock | 20-25% | moderate |
| TTRes | BO | Typic Orthic Brown Soils | Kake_1.1 | Kakepuku hill soils | Clayey | Tephra | 20-25% | moderate |
