# Supplementary material for: Agricultural Intensification Exacerbates Spillover Effects on Soil Biogeochemistry in Adjacent Forest Remnants
Source: PLoS One. 2015 Jan 9;10(1):e0116474. doi: 10.1371/journal.pone.0116474 (PMC4289067; doi:10.1371/journal.pone.0116474)
Supplement: S4 Table — ‘F’ and ‘U’ site codes indicate whether the adjacent forest remnants were fenced or unfenced. (DOCX) [file pone.0116474.s005.docx]

**Table S4.** Covariate effects tested in the mixed effects models for the influence of agricultural land-use intensity on farms surrounding the 21 forest remnants. ‘F’ and ‘U’ site codes indicate whether the adjacent forest remnants were fenced or unfenced.

| **Site code** | **Patch area** (ha) | **Pasture slope**  (°) | **Forest slope**  **(°)** | **Index of ‘recent change in intensity’** | **Land-use intensity** (PCA axis 1) |
| --- | --- | --- | --- | --- | --- |
| F1 | 4 | 10 | 25 | -0.4794 | 9.9379 |
| F2 | 6 | 3 | 5 | 3.0796 | 7.0991 |
| F3 | 3 | 14 | 22 | -1.9799 | 6.5701 |
| F4 | 9 | 32 | 13 | -1.5803 | 5.9754 |
| F5 | 3 | 23 | 17 | -1.2938 | 5.1850 |
| F6 | 4 | 4 | 3 | -0.0097 | 4.2685 |
| F7 | 8 | 15 | 20 | 0.9613 | 3.0668 |
| F8 | 4 | 16 | 11 | 0.3514 | 2.9150 |
| F9 | 10 | 5 | 14 | -1.4665 | 2.4971 |
| F10 | 5 | 10 | 21 | 3.3186 | 1.3721 |
| F11 | 16 | 8 | 20 | -1.1168 | 0.5014 |
| U1 | 3 | 3 | 17 | 0.0509 | 6.2200 |
| U2 | 4 | 20 | 19 | -1.3766 | 4.6877 |
| U3 | 2 | 18 | 22 | -0.8898 | 4.1473 |
| U4 | 10 | 16 | 15 | -0.7302 | 3.5691 |
| U5 | 2 | 20 | 15 | 1.2327 | 3.4113 |
| U6 | 4 | 16 | 19 | 1.7226 | 3.2730 |
| U7 | 7 | 15 | 14 | -0.1585 | 2.3228 |
| U8 | 2 | 6 | 19 | 3.3273 | 2.3867 |
| U9 | 3 | 17 | 13 | 3.1448 | 1.9667 |
| U10 | 16 | 12 | 15 | -0.7481 | 1.5680 |
