# Supplementary material for: Agricultural Intensification Exacerbates Spillover Effects on Soil Biogeochemistry in Adjacent Forest Remnants
Source: PLoS One. 2015 Jan 9;10(1):e0116474. doi: 10.1371/journal.pone.0116474 (PMC4289067; doi:10.1371/journal.pone.0116474)
Supplement: S5 Table — The eigenvectors give the coefficients for the linear combination of variables which defines the PCA axis. (DOCX) [file pone.0116474.s006.docx]

**Table S5.** Eigenvectors for the factor correlation matrix in the PCA ordination. The eigenvectors give the coefficients for the linear combination of variables which defines the PCA axis.

|  | **PC1** | **PC2** |
| --- | --- | --- |
| **Farmer inputs:** |  |  |
| N input | **0.340** | -0.187 |
| P input | 0.253 | -0.003 |
| Lime input | -0.132 | -0.065 |
| Stocking rate | 0.277 | **-0.320** |
|  |  |  |
| **Soil biogeochemistry:** |  |  |
| pH | -0.086 | **0.535** |
| Olsen P | **0.335** | -0.228 |
| Total C | 0.312 | **0.373** |
| Total N | 0.313 | **0.394** |
| C:N ratio | 0.028 | -0.071 |
| δ^15^N | 0.133 | **-0.414** |
| Total P | **0.400** | 0.105 |
| Total Cd | **0.332** | 0.187 |
| Total U | **0.350** | -0.058 |
