# Supplementary material for: Agricultural Intensification Exacerbates Spillover Effects on Soil Biogeochemistry in Adjacent Forest Remnants
Source: PLoS One. 2015 Jan 9;10(1):e0116474. doi: 10.1371/journal.pone.0116474 (PMC4289067; doi:10.1371/journal.pone.0116474)
Supplement: S6 Table — Values for ‘Farm area’ only represent the farm adjacent to the sampling sites, but larger forest reserves are surrounding by many other farms as well. All soil measures are the average of three recorded values taken in open pasture 46.5 m outside the forest edge at each site. (DOCX) [file pone.0116474.s007.docx]

**Table S6.** Components of variation in agricultural land-use intensity on farms surrounding the three forest reference sites, Maungakawa Reserve (MKRes; 965 ha), Te Miro Scenic Reserve (TMRes; 403 ha) and Te Tapui Reserve (TTRes; 1377 ha). Values for ‘Farm area’ only represent the farm adjacent to the sampling sites, but larger forest reserves are surrounding by many other farms as well. All soil measures are the average of three recorded values taken in open pasture 46.5 m outside the forest edge at each site.

| **Site code** | **Farm area**  ha | **Bulk density**  g.cm^-3^ | **Moisture factor** | **N input** kg.ha^-1^. yr^-1^ | **P input** kg.ha^-1^. yr^-1^ | **Lime input**  t.ha^-1^.yr^-1^ | **Stocking rate** SU.ha^-1^ | **pH** | **Olsen P** μg.cm^-3^ | **Total C** mg.cm^-3^ | **Total N** mg.cm^-3^ | **C:N**  **ratio** | **δ^15^N**  ‰ | **Total P**  μg.cm^-3^ | **Total Cd**  μg.cm^-3^ | **Total U**  μg.cm^-3^ |
| --- | --- | --- | --- | --- | --- | --- | --- | --- | --- | --- | --- | --- | --- | --- | --- | --- |
| MKRes | 357 | 0.514 | 1.106 | 68.0 | 0.0 | 0.0 | 11.1 | 5.18 | 6.61 | 56.39 | 5.63 | 10.03 | 5.09 | 706.68 | 0.30 | 0.92 |
| TMRes | 404 | 0.623 | 1.105 | 101.8 | 31.9 | 0.2 | 12.4 | 5.42 | 4.92 | 57.88 | 6.12 | 9.46 | 4.99 | 779.81 | 0.30 | 1.26 |
| TTRes | 404 | 0.764 | 1.053 | 72.0 | 43.4 | 0.0 | 12.5 | 5.42 | 28.07 | 45.15 | 4.88 | 9.27 | 5.26 | 939.63 | 0.54 | 1.44 |
