# Supplementary material for: Agricultural Intensification Exacerbates Spillover Effects on Soil Biogeochemistry in Adjacent Forest Remnants
Source: PLoS One. 2015 Jan 9;10(1):e0116474. doi: 10.1371/journal.pone.0116474 (PMC4289067; doi:10.1371/journal.pone.0116474)
Supplement: S7 Table — (DOCX) [file pone.0116474.s008.docx]

**Table S7.** Results of mixed-effects modelling of the effects of land-use intensity on gravimetric measures of soil nutrient geochemistry in

native forest remnants embedded within production landscapes. AIC, Akaike Information Criterion; PCV, proportion change in variance; VC, variance components. For full models, the intercept represents the edge of unfenced forest remnants at low surrounding land-use intensity. Model parameters were calculated using restricted maximum likelihood (REML) estimation, and a weighted model averaging approach. Bolded coefficients are significantly different from zero (P<0.05).

| **Response variable:** | **Total carbon (%)** | |  | **Total Nitrogen (%)** | |  | **Olsen P (mg.kg^-1^)** | |  | **Total P (mg.kg^-1^)** | |
| --- | --- | --- | --- | --- | --- | --- | --- | --- | --- | --- | --- |
|  | **Null model** | **Full model** |  | **Null model** | **Full model** |  | **Null model** | **Full model** |  | **Null model** | **Full model** |
| Fixed effects | *b* [±1 SE] | *b* [±1 SE] |  | *b*  [±1 SE] | *b* [±1 SE] |  | *b* [±1 SE] | *b* [±1 SE] |  | *b* [±1 SE] | *b*  [±1 SE] |
| Intercept | **-3.238 [0.558]** | **-3.007 [0.700]** |  | **-0.163 [0.050]** | -0.084 [0.073] |  | **2.258 [0.201]** | **2.943 [0.260]** |  | **0.407 [0.076]** | **0.725 [0.124]** |
| Pasture slope | - | -1.189 [1.126] |  | - | -0.066 [0.090] |  | - | 0.212 [0.267] |  | - | -0.169 [0.127] |
| Forest slope | - | -1.041 [1.004] |  | - | -0.139 [0.087] |  | - | **0.681 [0.258]** |  | - | -0.151 [0.130] |
| Patch area | - | 1.404 [1.092] |  | - | 0.063 [0.095] |  | - | - |  | - | - |
| Recent change in intensity | - | -1.598 [1.077] |  | - | -0.101 [0.090] |  | - | **0.948 [0.264]** |  | - | 0.164 [0.134] |
|  |  |  |  |  |  |  |  |  |  |  |  |
| Land-use intensity gradient | - | 3.047 [2.668] |  | - | 0.214 [0.125] |  | - | 0.236 [0.262] |  | - | 0.740 [0.434] |
| Fencing | - | -0.746 [1.057] |  | - | -0.103 [0.091] |  | - | -0.755 [0.362] |  | - | -0.335 [0.161] |
| Distance from edge (linear) | - | -0.808 [0.487] |  | - | **-0.100 [0.038]** |  | - | -0.411 [0.291] |  | - | **-0.344 [0.105]** |
| Distance from edge (quadratic) | - | -1.583 [1.108] |  | - | -0.116 [0.079] |  | - | -0.671 [0.371] |  | - | -0.319 [0.194] |
| Land-use : Fencing | - | **-5.665 [2.516]** |  | - | -0.329 [0.220] |  | - | - |  | - | -0.867 [0.413] |
| Land-use : Distance (linear) | - | - |  | - | - |  | - | -0.687 [0.361] |  | - | **-0.868 [0.303]** |
| Land-use : Distance (quad.) | - | - |  | - | - |  | - | -0.020 [0.550] |  | - | **-1.159 [0.563]** |
| Fencing : Distance (linear) | - | - |  | - | - |  | - | - |  | - | -0.205 [0.139] |
| Fencing : Distance (quad) | - | - |  | - | - |  | - | - |  | - | 0.146 [0.251] |
| Land-use : Fencing : Dist(lin) | - | - |  | - | - |  | - | - |  | - | **1.116 [0.336]** |
| Land-use : Fencing:Dist(quad) | - | - |  | - | - |  | - | - |  | - | **1.654 [0.624]** |
|  |  |  |  |  |  |  |  |  |  |  |  |
| VC for random effects | VC | VC |  | VC | VC |  | VC | VC |  | VC | VC |
| Site | 9.71590 | 9.00146 |  | 0.07574 | 0.07298 |  | 1.19578 | 0.66073 |  | 0.15107 | 0.12398 |
| Random edge effect | 18.73671 | 18.34312 |  | 0.09912 | 0.08451 |  | 2.11485 | 1.21699 |  | 0.31844 | 0.09544 |
| Distance block within site | 1.96667 | 1.97983 |  | 0.01124 | 0.01124 |  | 0.14128 | 0.14580 |  | 0.04366 | 0.04269 |
| Residuals | 0.99121 | 0.99149 |  | 0.00476 | 0.00476 |  | 0.10509 | 0.10505 |  | 0.02334 | 0.02334 |
| VC for fixed effects | - | 0.92419 |  | - | 0.01347 |  | - | 0.64998 |  | - | 0.08073 |
|  |  |  |  |  |  |  |  |  |  |  |  |
| PCV_[Site]_ | - | 7.35% |  | - | 3.64% |  | - | 44.74% |  | - | 17.93% |
| PCV_[Edge.Slope]_ | - | 2.10% |  | - | 14.74% |  | - | 42.46% |  | - | 70.03% |
| PCV_[Edge.Block]_ | - | -0.67% |  | - | 0.00% |  | - | -3.20% |  | - | 2.22% |
| PCV_[Residuals]_ | - | -0.03% |  | - | 0.00% |  | - | 0.03% |  | - | -0.01% |
|  |  |  |  |  |  |  |  |  |  |  |  |
| $R_{GLMM(m)}^{2}$ | - | 2.96% |  | - | 7.15% |  | - | 23.37% |  | - | 22.47% |
| $R_{GLMM(c)}^{2}$ | - | 96.82% |  | - | 97.45% |  | - | 96.22% |  | - | 93.55% |
| AIC | 1192.860 | 1189.581 |  | -454.245 | -460.998 |  | 485.389 | 466.007 |  | 21.417 | 3.084 |
| **Response variable:** | **Total Cd (mg.kg^-1^)** | |  | **Total U (mg.kg^-1^)** | |  |  | |  |  | |
|  | **Null model** | **Full model** |  | **Null model** | **Full model** |  |  |  |  |  |  |
| Fixed effects | *b*  [±1 SE] | *b* [±1 SE] |  | *b* [±1 SE] | *b* [±1 SE] |  |  |  |  |  |  |
| Intercept | **0.146 [0.018]** | **0.177 [0.027]** |  | **0.171 [0.054]** | **0.201 [0.055]** |  |  |  |  |  |  |
| Pasture slope |  | -0.038 [0.028] |  | - | **-0.124 [0.057]** |  |  |  |  |  |  |
| Forest slope |  | -0.040 [0.030] |  | - | **0.189 [0.057]** |  |  |  |  |  |  |
| Patch area |  | **0.074 [0.035]** |  | - | 0.090 [0.067] |  |  |  |  |  |  |
| Recent change in intensity | - | 0.043 [0.032] |  | - | **-0.286 [0.069]** |  |  |  |  |  |  |
|  |  |  |  |  |  |  |  |  |  |  |  |
| Land-use intensity gradient | - | **0.085 [0.036]** |  | - | -0.316 [0.160] |  |  |  |  |  |  |
| Fencing | - | **-0.076 [0.032]** |  | - | **-0.132 [0.060]** |  |  |  |  |  |  |
| Distance from edge (linear) | - | **-0.062 [0.018]** |  | - | -0.085 [0.045] |  |  |  |  |  |  |
| Distance from edge (quadratic) | - | 0.057 [0.034] |  | - | 0.015 [0.068] |  |  |  |  |  |  |
| Land-use : Fencing | - | - |  | - | **0.483 [0.150]** |  |  |  |  |  |  |
| Land-use : Distance (linear) | - | - |  | - | **-0.255 [0.091]** |  |  |  |  |  |  |
| Land-use : Distance (quad.) | - | - |  | - | 0.186 [0.135] |  |  |  |  |  |  |
| Fencing : Distance (linear) | - | - |  | - | - |  |  |  |  |  |  |
| Fencing : Distance (quadratic) | - | - |  | - | - |  |  |  |  |  |  |
| Land-use : Fencing : Dist(lin.) | - | - |  | - | - |  |  |  |  |  |  |
| Land-use : Fencing:Dist(quad) | - | - |  | - | - |  |  |  |  |  |  |
|  |  |  |  |  |  |  |  |  |  |  |  |
| VC for random effects | VC | VC |  | VC | VC |  |  |  |  |  |  |
| Site | 0.00700 | 0.00617 |  | 0.06487 | 0.03689 |  |  |  |  |  |  |
| Random edge effect | 0.02157 | 0.01596 |  | 0.05744 | 0.03880 |  |  |  |  |  |  |
| Distance block within site | 0.00165 | 0.00165 |  | 0.01328 | 0.01321 |  |  |  |  |  |  |
| Residuals | 0.00234 | 0.00234 |  | 0.00787 | 0.00787 |  |  |  |  |  |  |
| VC for fixed effects | - | 0.00325 |  | - | 0.05022 |  |  |  |  |  |  |
|  |  |  |  |  |  |  |  |  |  |  |  |
| PCV_[Site]_ | - | 11.81% |  | - | 43.14% |  |  |  |  |  |  |
| PCV_[Edge.Slope]_ | - | 26.01% |  | - | 32.45% |  |  |  |  |  |  |
| PCV_[Edge.Block]_ | - | -0.01% |  | - | 0.49% |  |  |  |  |  |  |
| PCV_[Residuals]_ | - | 0.01% |  | - | 0.01% |  |  |  |  |  |  |
|  |  |  |  |  |  |  |  |  |  |  |  |
| $R_{GLMM(m)}^{2}$ | - | 11.02% |  | - | 34.26% |  |  |  |  |  |  |
| $R_{GLMM(c)}^{2}$ | - | 92.03% |  | - | 94.62% |  |  |  |  |  |  |
| AIC | -768.327 | -784.391 |  | -323.918 | -351.442 |  |  |  |  |  |  |
