# Supplementary material for: Agricultural Intensification Exacerbates Spillover Effects on Soil Biogeochemistry in Adjacent Forest Remnants
Source: PLoS One. 2015 Jan 9;10(1):e0116474. doi: 10.1371/journal.pone.0116474 (PMC4289067; doi:10.1371/journal.pone.0116474)
Supplement: S1 Fig — Variables were transformed where necessary (see text) and normalised for subsequent PCA ordination analysis. Values above the diagonal are Pearson correlation coefficients, size-scaled by the value of the coefficient. (PDF) [file pone.0116474.s009.pdf]

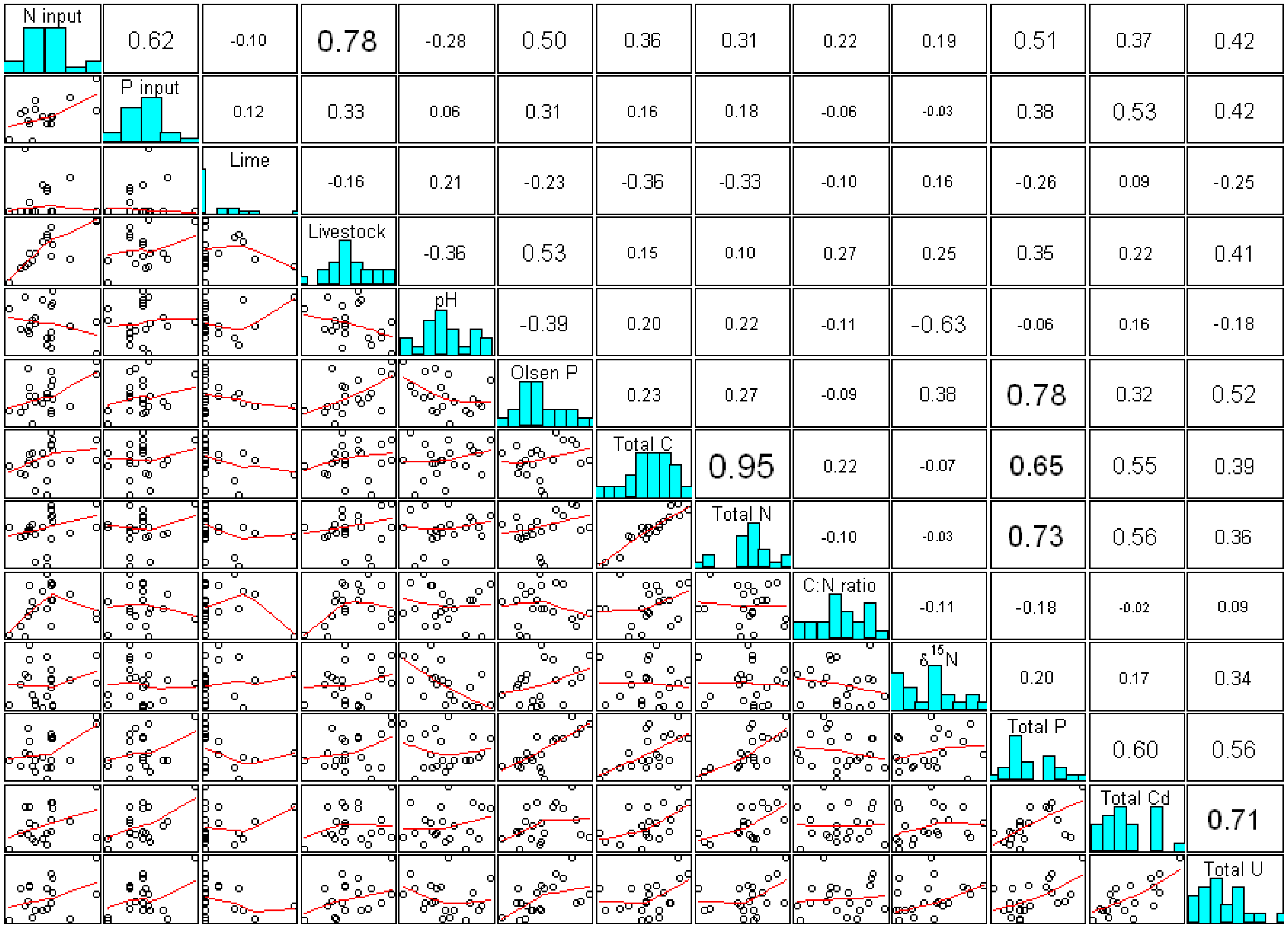

Figure S1. Correlation matrix for the 13 measures of agricultural land-use intensity on farms surrounding the 21 forest remnants. Variables were transformed where necessary (see text) and normalised for subsequent PCA ordination analysis. Values above the diagonal are Pearson correlation coefficients, size-scaled by the value of the coefficient.
