# Supplementary material for: Agricultural Intensification Exacerbates Spillover Effects on Soil Biogeochemistry in Adjacent Forest Remnants
Source: PLoS One. 2015 Jan 9;10(1):e0116474. doi: 10.1371/journal.pone.0116474 (PMC4289067; doi:10.1371/journal.pone.0116474)
Supplement: S2 Fig — (PDF) [file pone.0116474.s010.pdf]

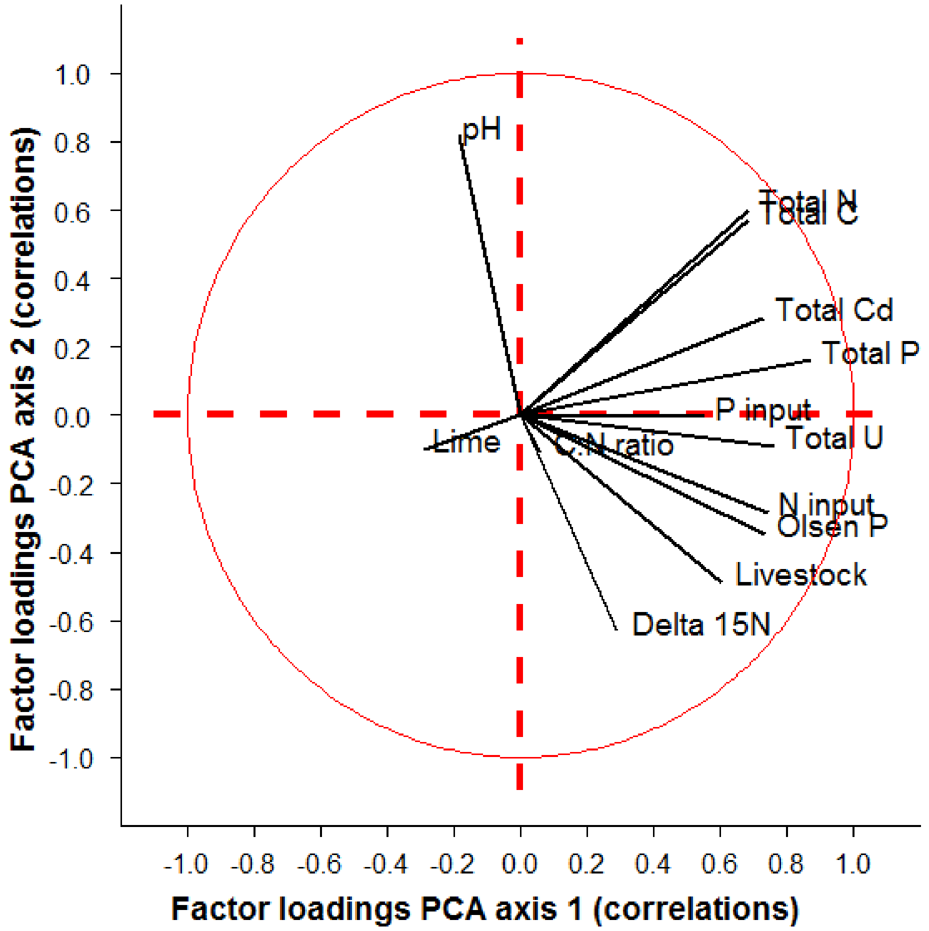

Figure S2. Factor loadings (correlations) on PCA axes 1 and 2 showing the dominant farmer input measures and soil biogeochemistry measures driving variation in land-use intensity across farms.
