# Supplementary material for: Agricultural Intensification Exacerbates Spillover Effects on Soil Biogeochemistry in Adjacent Forest Remnants
Source: PLoS One. 2015 Jan 9;10(1):e0116474. doi: 10.1371/journal.pone.0116474 (PMC4289067; doi:10.1371/journal.pone.0116474)
Supplement: S3 Fig — See text for explanation. (PDF) [file pone.0116474.s011.pdf]

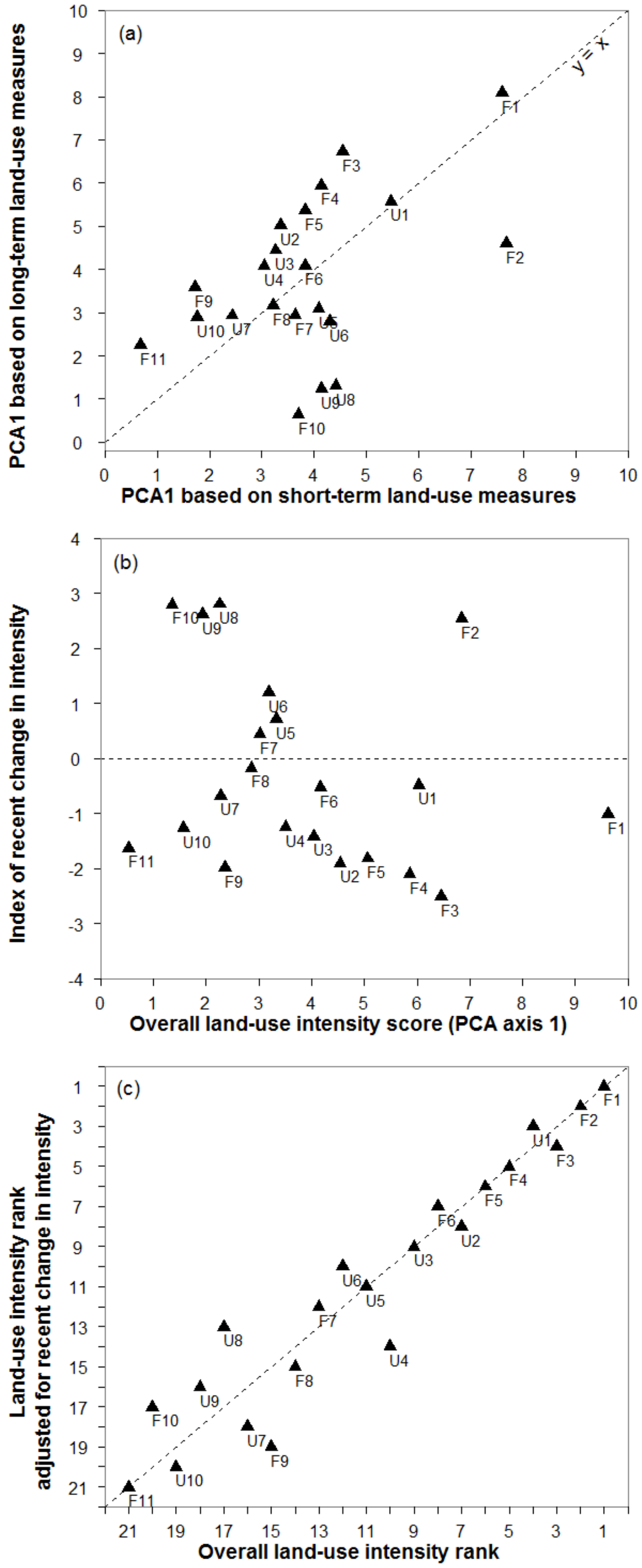

Figure S3. Sensitivity test of the influence of using short-term versus longer-term land-use intensity measures to derive a composite overall land-use intensity gradient. See text for explanation.
