# Supplementary material for: Agricultural Intensification Exacerbates Spillover Effects on Soil Biogeochemistry in Adjacent Forest Remnants
Source: PLoS One. 2015 Jan 9;10(1):e0116474. doi: 10.1371/journal.pone.0116474 (PMC4289067; doi:10.1371/journal.pone.0116474)
Supplement: S4 Fig — No consistent clustering or confounding effect of soil type was evident along the PCA axis 1 land-use intensity gradient. (PDF) [file pone.0116474.s012.pdf]

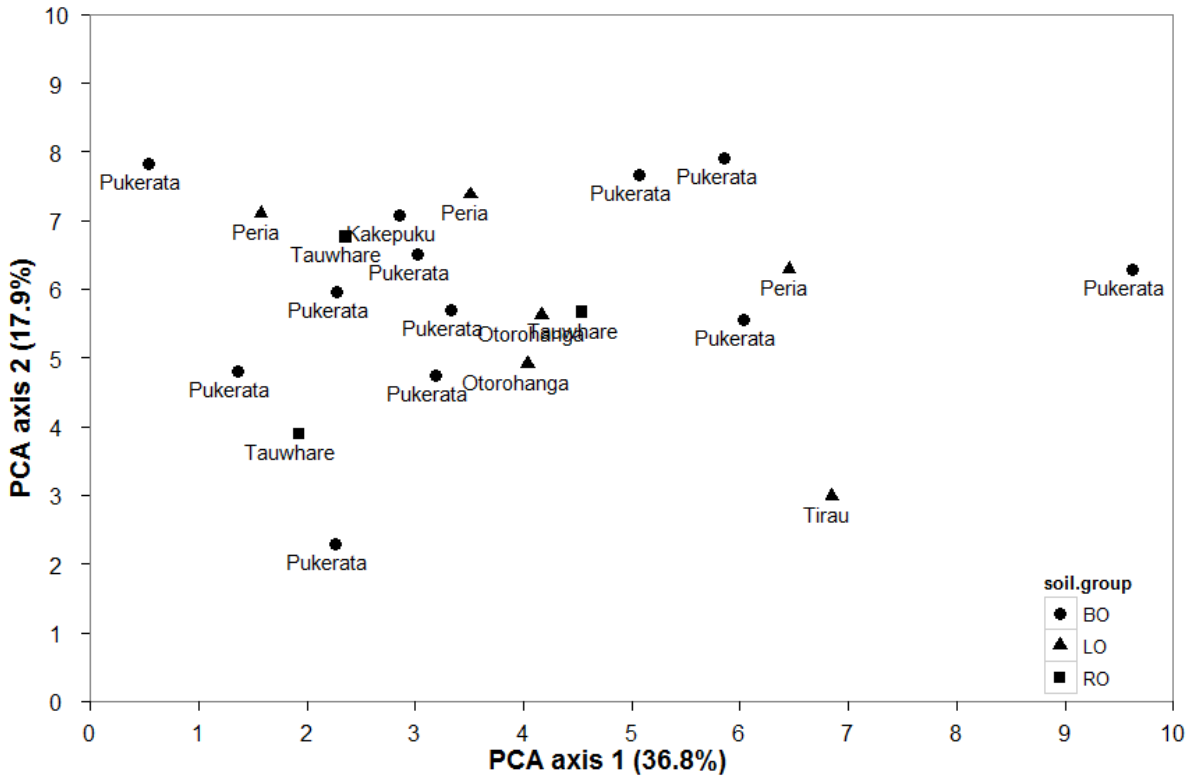

Figure S4. The biplot of PCA axes 1 and 2 from Figure 2 (based on all 13 farmer input and soil biogeochemistry measures) re-plotted to show variation in soil groups (symbols: BO = Typic Orthic Brown Soils; LO = Typic Orthic Allophanic Soils; RO = Mottled Orthic Recent Soils) and names of soil types for each farm. No consistent clustering or confounding effect of soil type was evident along the PCA axis 1 land-use intensity gradient.
