# Supplementary material for: Agricultural Intensification Exacerbates Spillover Effects on Soil Biogeochemistry in Adjacent Forest Remnants
Source: PLoS One. 2015 Jan 9;10(1):e0116474. doi: 10.1371/journal.pone.0116474 (PMC4289067; doi:10.1371/journal.pone.0116474)
Supplement: S5 Fig — The mean (± 95 confidence limits) for Moran’s I indicate whether the observed pairwise similarity at a given scale is significantly greater or less than expected by chance alone, using a resampling procedure with 1000 random draws in the ncf package in R2.14.2. (PDF) [file pone.0116474.s013.pdf]

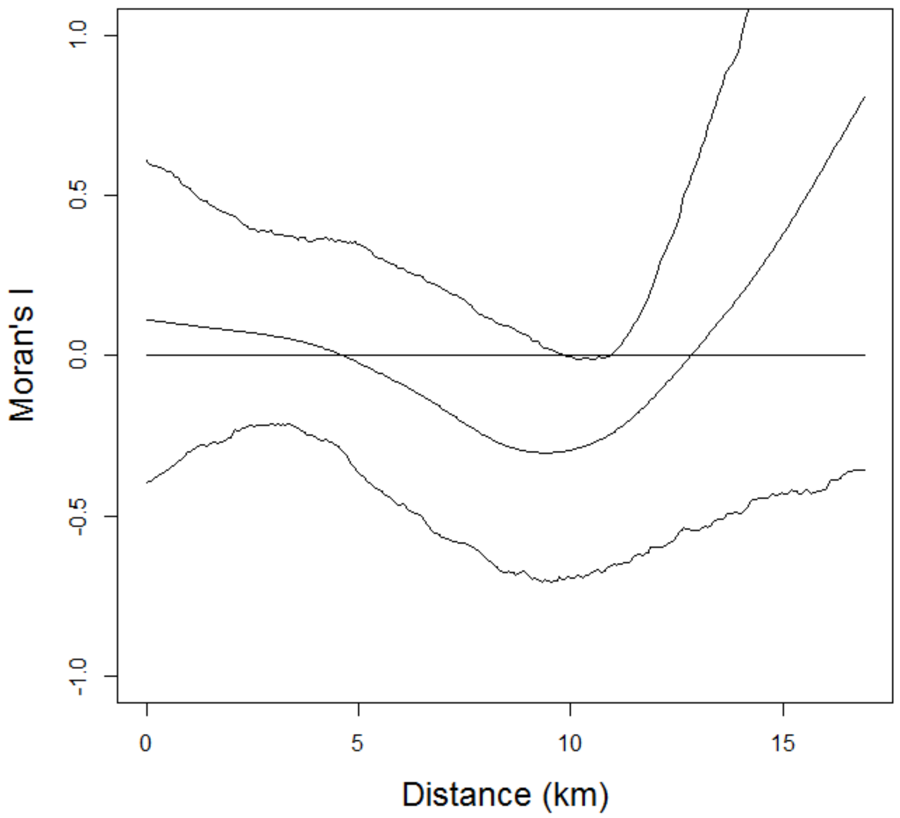

Figure S5. Spline correlogram showing whether the spatial location of sampling sites in the landscape had a significant effect on the pairwise similarity of PCA axis 1 land-use intensity scores among sites. The mean (± 95 confidence limits) for Moran's I indicate whether the observed pairwise similarity at a given scale is significantly greater or less than expected by chance alone, using a resampling procedure with 1000 random draws in the ncf package in R2.14.2.
