# Supplementary material for: Agricultural Intensification Exacerbates Spillover Effects on Soil Biogeochemistry in Adjacent Forest Remnants
Source: PLoS One. 2015 Jan 9;10(1):e0116474. doi: 10.1371/journal.pone.0116474 (PMC4289067; doi:10.1371/journal.pone.0116474)
Supplement: S6 Fig — See text for further explanation. (PDF) [file pone.0116474.s014.pdf]

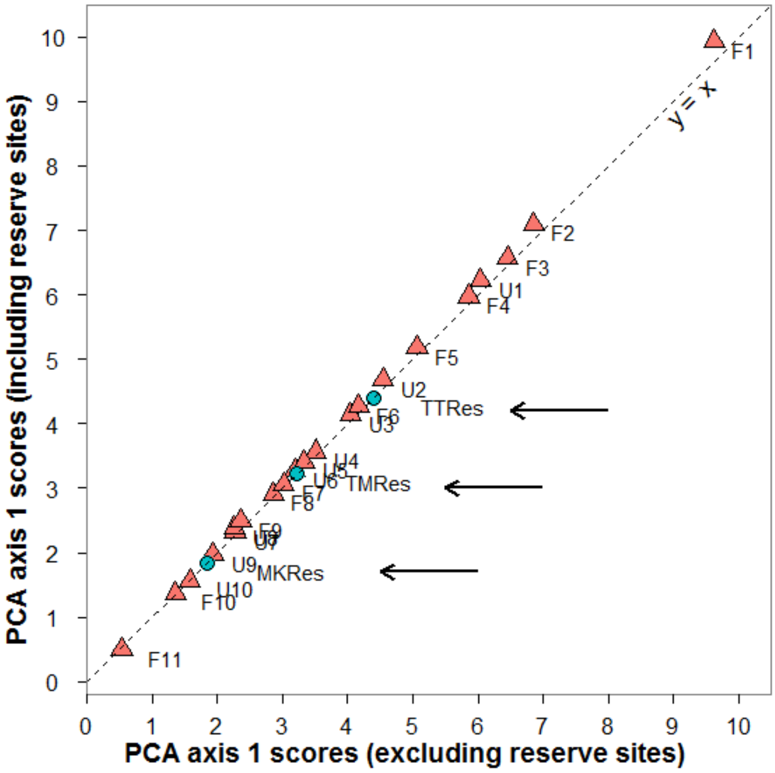

Figure S6. Comparative analysis of where farms adjacent to the three forest reference sites (blue circles) were placed along the composite land-use intensity gradient for farms surrounding the 21 forest remnant sites (red triangles). See text for further explanation.
