# Supplementary material for: Agricultural Intensification Exacerbates Spillover Effects on Soil Biogeochemistry in Adjacent Forest Remnants
Source: PLoS One. 2015 Jan 9;10(1):e0116474. doi: 10.1371/journal.pone.0116474 (PMC4289067; doi:10.1371/journal.pone.0116474)
Supplement: S7 Fig — The mean (± 1 S.E.) fitted relationships were derived from the final model-averaged solution (Table 2) using the ‘lmePredict’ function, while holding other fixed effects constant at their mean values. Symbols represent the raw means (± 1 S.E.). The grey shaded interval represents the raw mean (± 1 S.E.) of the soil moisture factor at the reference forest interior sites (243–420 m from edge). Note that the predicted relationships are conditional on the random effects specified in the model. (PDF) [file pone.0116474.s015.pdf]

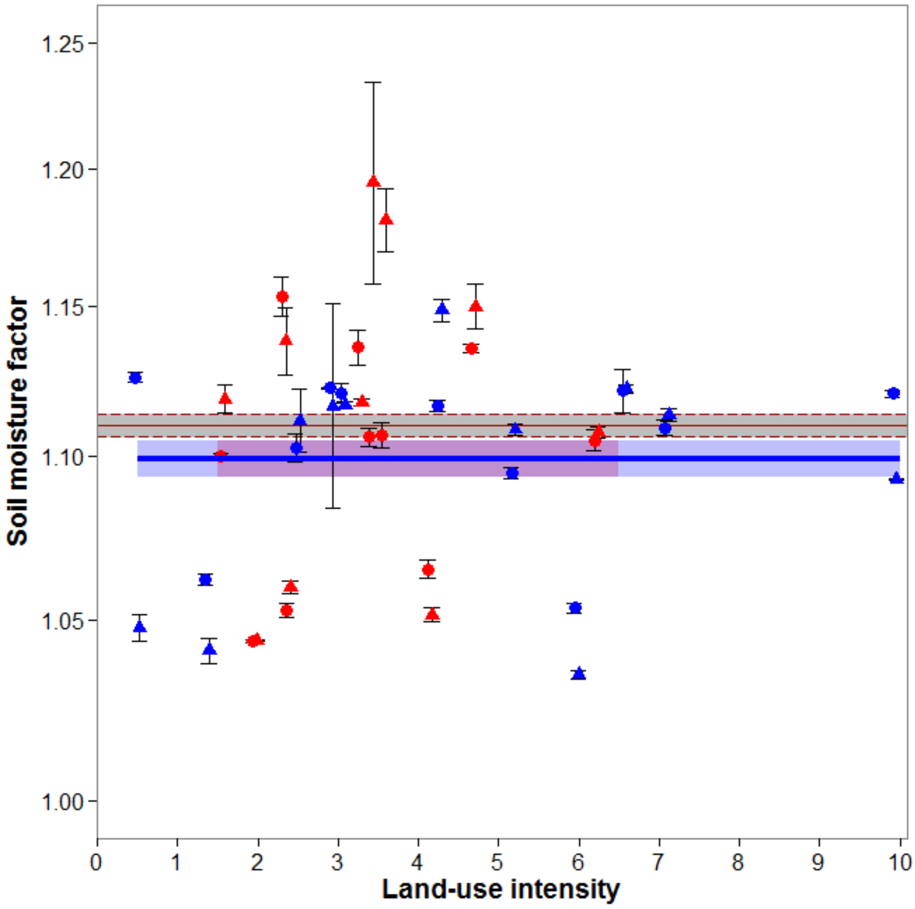

Figure S7. Predicted relationships between surrounding agricultural land-use intensity and soil moisture factor within fenced and unfenced forest remnants. The mean (± 1 S.E.) fitted relationships were derived from the final model-averaged solution (Table 2) using the 'lmePredict' function, while holding other fixed effects constant at their mean values. Symbols represent the raw means (± 1 S.E.). The grey shaded interval represents the raw mean (± 1 S.E.) of the soil moisture factor at the reference forest interior sites (243 – 420 m from edge). Note that the predicted relationships are conditional on the random effects specified in the model.
