# Supplementary material for: Agricultural Intensification Exacerbates Spillover Effects on Soil Biogeochemistry in Adjacent Forest Remnants
Source: PLoS One. 2015 Jan 9;10(1):e0116474. doi: 10.1371/journal.pone.0116474 (PMC4289067; doi:10.1371/journal.pone.0116474)
Supplement: S8 Fig — See Fig. 4 for details. (PDF) [file pone.0116474.s016.pdf]

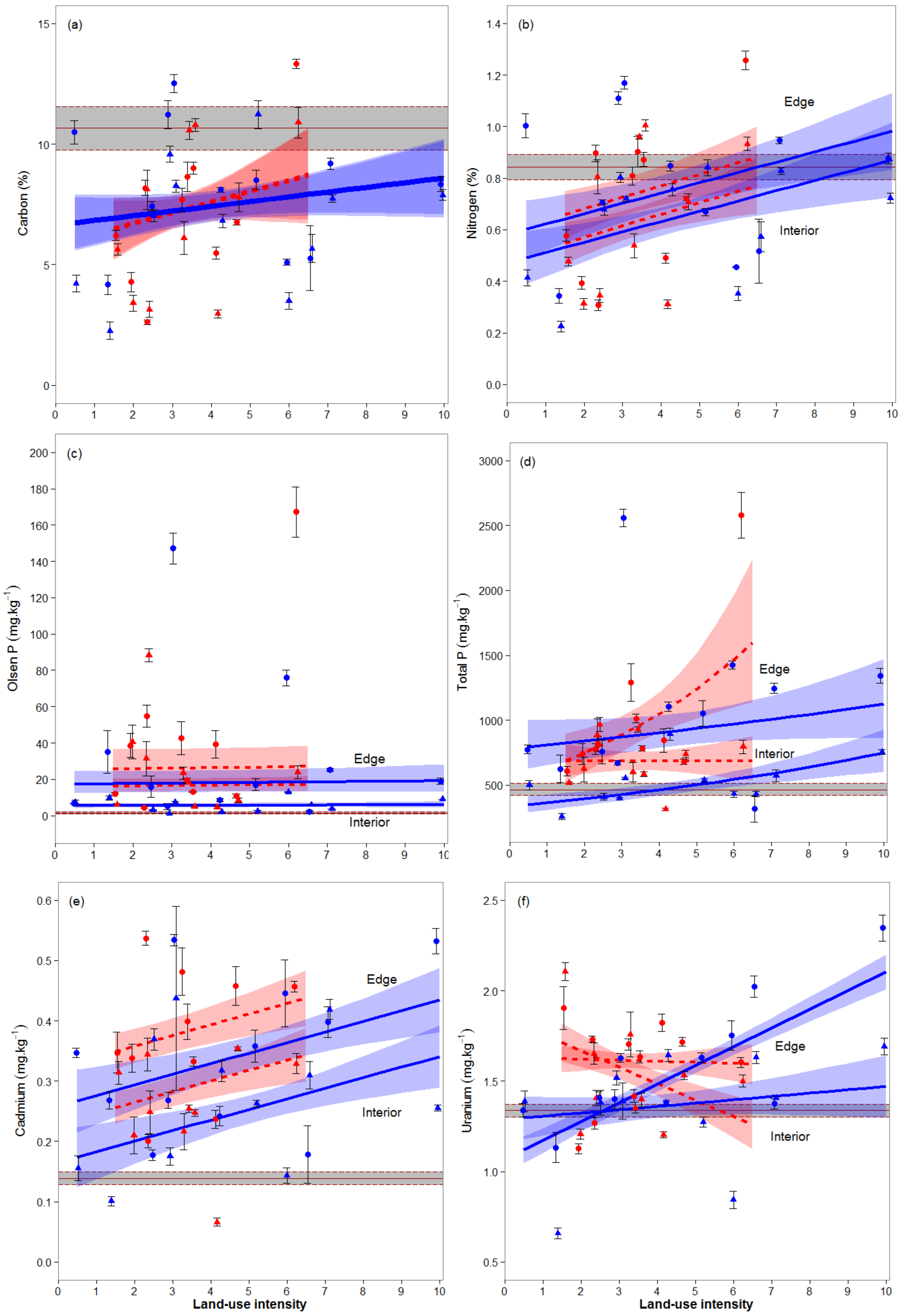

(a)
Carbon (%)
(b)
Nitrogen (%)
Edge
Interior
(c)
Olsen P (mg.kg^{-1})
Edge
Interior
(d)
Total P (mg.kg^{-1})
Edge
Interior
(e)
Cadmium (mg.kg^{-1})
Edge
Interior
(f)
Uranium (mg.kg^{-1})
Edge
Interior
Land-use intensity
Land-use intensity

Figure S8 (*preceding page*). Predicted relationships between surrounding agricultural land-use intensity and soil biogeochemistry within fenced and unfenced forest remnants, re-analysed and plotted as gravimetric response measures rather than as volumetric response measures. See Figure 4 for details.
